# Mending a broken heart by biomimetic 3D printed natural biomaterial-based cardiac patches: a review

**DOI:** 10.3389/fbioe.2023.1254739

**Published:** 2023-11-16

**Authors:** Elisabetta Rosellini, Maria Grazia Cascone, Lorenzo Guidi, Dirk W. Schubert, Judith A. Roether, Aldo R. Boccaccini

**Affiliations:** ^1^ Department of Civil and Industrial Engineering, University of Pisa, Pisa, Italy; ^2^ Department of Materials Science and Engineering, Institute of Polymer Materials, Friedrich-Alexander-University (FAU), Erlangen, Germany; ^3^ Bavarian Polymer Institute (BPI), Erlangen, Germany; ^4^ Department of Materials Science and Engineering, Institute of Biomaterials, Friedrich-Alexander-University (FAU), Erlangen, Germany

**Keywords:** 3D printing, 3D bioprinting, bioink, natural biomaterials, cardiac tissue engineering, biomimicry, functionalization

## Abstract

Myocardial infarction is one of the major causes of mortality as well as morbidity around the world. Currently available treatment options face a number of drawbacks, hence cardiac tissue engineering, which aims to bioengineer functional cardiac tissue, for application in tissue repair, patient specific drug screening and disease modeling, is being explored as a viable alternative. To achieve this, an appropriate combination of cells, biomimetic scaffolds mimicking the structure and function of the native tissue, and signals, is necessary. Among scaffold fabrication techniques, three-dimensional printing, which is an additive manufacturing technique that enables to translate computer-aided designs into 3D objects, has emerged as a promising technique to develop cardiac patches with a highly defined architecture. As a further step toward the replication of complex tissues, such as cardiac tissue, more recently 3D bioprinting has emerged as a cutting-edge technology to print not only biomaterials, but also multiple cell types simultaneously. In terms of bioinks, biomaterials isolated from natural sources are advantageous, as they can provide exceptional biocompatibility and bioactivity, thus promoting desired cell responses. An ideal biomimetic cardiac patch should incorporate additional functional properties, which can be achieved by means of appropriate functionalization strategies. These are essential to replicate the native tissue, such as the release of biochemical signals, immunomodulatory properties, conductivity, enhanced vascularization and shape memory effects. The aim of the review is to present an overview of the current state of the art regarding the development of biomimetic 3D printed natural biomaterial-based cardiac patches, describing the 3D printing fabrication methods, the natural-biomaterial based bioinks, the functionalization strategies, as well as the *in vitro* and *in vivo* applications.

## 1 Introduction

Cardiovascular diseases are the leading cause of mortality as well as morbidity worldwide (WHO, 2016). Among cardiovascular diseases, myocardial infarction (MI) is a dominant medical problem. It consists of a partial or complete occlusion of one of the coronary arteries, which causes either a reduction or a stop of the blood supply to the heart, thereby causing necrosis of the cardiac tissue (Thygesen et al., 2007). Adult cardiac tissue is not able to repair itself due to the limited regenerative capacity of cardiomyocytes (CMs) in the post-natal life. Therefore, the necrotic cardiac muscle is substituted by a scar tissue, which is not able to conduct electrical and mechanical stimuli, thus leading to an abnormal contractility of the heart and subsequently to progressive heart remodeling and heart failure (Jhund and McMurray, 2008; Minicucci et al., 2011; Wang et al., 2018). Pharmacological approaches using drugs are the first treatment option offered to patients, but they can only provide a temporary improvement of the quality of life, without any capability to regenerate the diseased myocardium and consequently to alter the trajectory of the disease. In the later stages of heart failure, transplantation is the only treatment option. However, it presents serious limitations, such as the long waiting time, shortage of organ donors, high incidence of post-operative complications, the risk of immune rejection and the problems connected to a life-long immunosuppressive therapy. As such, there is an urgent need for alternative treatments.

Cardiac tissue engineering (CTE), through an appropriate combination of cells, scaffold and signals, aims to bioengineer new and functional cardiac tissue, which could be used for tissue repair, patient specific drug screening as well as disease modeling, thus representing a new prospect to treat one of the most fatal cardiovascular diseases (Zammaretti and Jaconi, 2004). Each element of the tissue engineering triad (cells, scaffold and signaling factors) has a profound influence on the satisfactory outcome of the tissue engineering procedure. In particular, in the last years, the importance of the development of a biomimetic scaffold, with structural and compositional features analogous to those of the native tissue, emerged as a key aspect.

**FIGURE 1:**
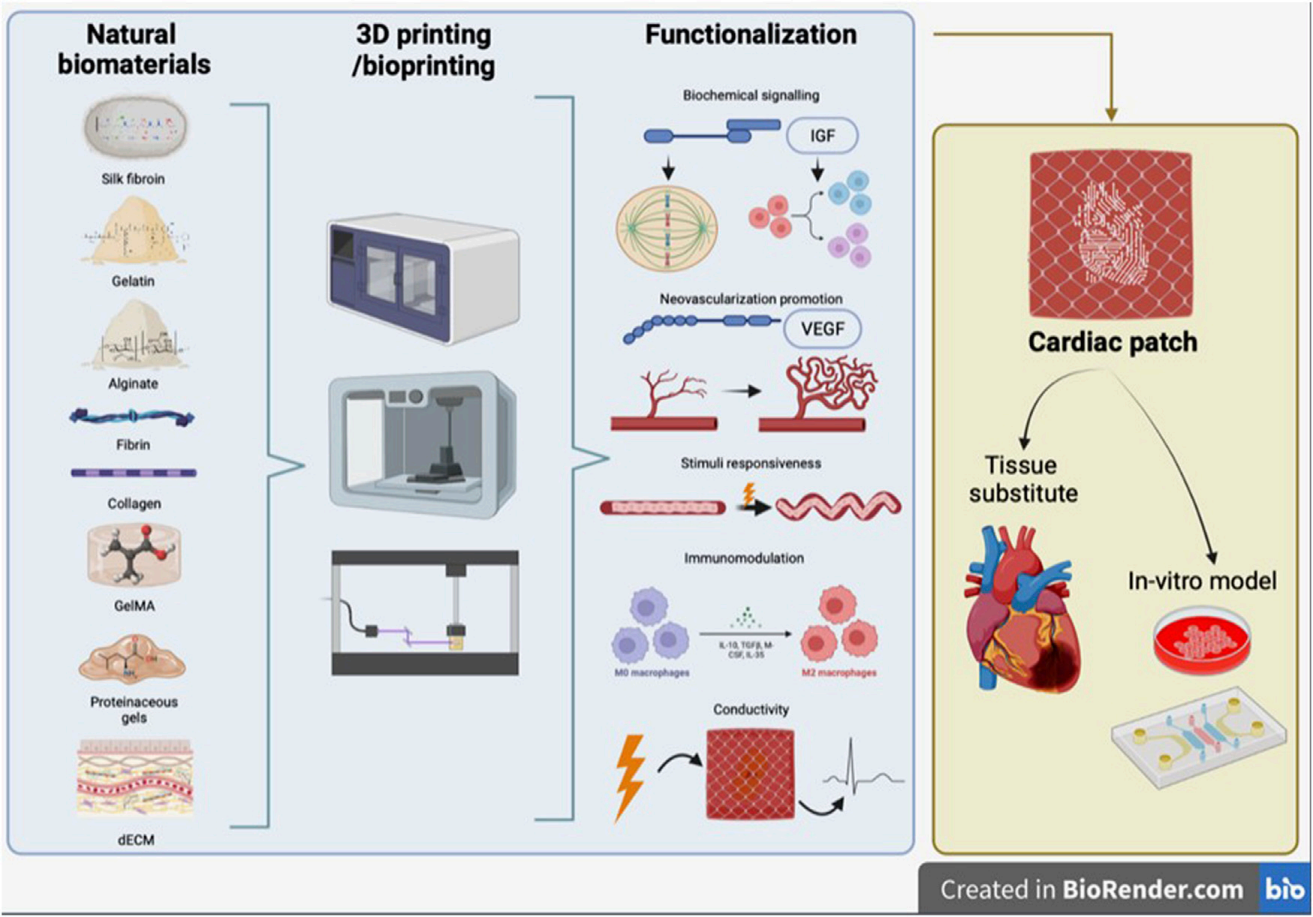
Schematic representation of review concept. Synergic combination of 3D printing/bioprinting with natural biomaterials and functionalization strategies, to produce a bioengineered cardiac tissue, which can be used both as tissue substitute and as *in vitro* model.

The development of a biomimetic scaffold comprises different aspects, such as the fabrication technique, which determines the macro- and microarchitecture of the scaffold, the material used for scaffold fabrication and the inclusion within the scaffold of bioactive molecules or other stimulating factors to promote the formation of a functional tissue (Figure 1).

Among scaffold fabrication techniques, three-dimensional (3D) printing has been recognized in recent years as a promising technique to develop a new generation of cardiac patches. This technique is an additive manufacturing technique, which involves the translation of computer-aided designs into 3D objects, by means of a very precise layer-by-layer approach. The accurate control over the printing process permits an effective replication of pivotal features of the native tissue. In particular, 3D printing enables the fabrication of scaffolds of complex architecture and with a spatio-temporal distribution of bioactive substances, to direct tissue growth and organization effectively. As a further step toward the replication of thick complex tissues, such as the cardiac one, more recently 3D bioprinting has been established as a cutting-edge technology to print not only biomaterials, but also multiple cell types, with unparalleled control over their positioning, hence achieving the overall replication of the structure and composition of the native tissue.

Different biomaterials, of either natural or synthetic origin as well as combinations of both, have been used as bioinks to produce cardiac patches by 3D printing/bioprinting. In the development of a biomimetic patch, biomaterials isolated from natural sources are advantageous, as they can provide exceptional biocompatibility, effective biological imitation of myocardial tissue, as well as adequate biochemical and physical properties to printed constructs, thus promoting desired cell behavior in terms of migration, proliferation, differentiation and maturation (Sharma et al., 2019; Majid et al., 2020).

In addition to appropriate chemical and mechanical properties (given by the biomaterial) and a suitable structure for cell alignment and elongation (given by scaffold architecture), an ideal biomimetic cardiac patch should also display additional functional properties that are essential to replicate the native tissue. The scaffold should be decorated with bioactive agents, such as growth factors, extracellular vesicles or immunomodulatory substances, to promote an effective regeneration process and prevent the formation of fibrous capsules as well as chronic inflammatory response. Moreover, the survival of the engineered patch should be favored by promoting neo-vascularization through the incorporation of vascular cells or pro-angiogenic factors. In addition, the integration with the surrounding contractile tissue and an adequate synchronous contraction ability can be achieved only developing a conductive matrix, able to conduct the bioelectric signals and avoid severe arrhythmia *in vivo*. More recently, advanced functional properties such as 4D smart patches and shape memory effects are also under investigation.

The purpose of the review is to present an overview of the current state of the art regarding the development of biomimetic 3D printed natural biomaterial-based cardiac patches. Starting from the physiological and anatomical features of the native cardiac extracellular matrix (ECM), we will then focus on three aspects: the 3D printing fabrication methods, the natural-polymer based bioinks and the functionalization strategies. We will then conclude illustrating the possible applications, both as *in vivo* replacement tissue and as *in vitro* tissue models, underlining their potential advantages with respect to more traditional CTE methods.

## 2 Native cardiac ECM: structure and properties

The heart is primarily composed of a striated muscle tissue known as the myocardium. This myocardium consists of two to four billion cells referred to as CMs, which have the responsibility for heart contraction and relaxation, by conducting electrical signals. While CMs make up 75% of the heart’s volume, they constitute only 30% of the total cell count. CMs are interconnected with various other cell types, forming a complex 3D structure (Zhang et al., 2022). Among the non-myocyte cells, there are endothelial cells, making up approximately 60% of the non-myocyte resident cells, forming an intricate network of capillaries that supply nutrients to the tissue. Additionally, there are smooth muscle cells, fibroblasts that produce the ECM for structural support, pericytes, and resident immune cells like macrophages, along with a small number of B and T cells. The cardiac muscle also contains specialized cells known as Purkinje cells, which are responsible for rapidly conducting electrical signals (Wang et al., 2021b). About a quarter of the cardiac volume is composed of the ECM, which plays a crucial role in shaping tissue architecture and maintaining the internal environment balance, by regulating interactions between cells and the surrounding matrix (Zent and Pozzi, 2010).

The cardiac ECM is an intricate system, comprising fibrous elements (such as collagen fibers) and non-fibrous elements (such as proteoglycans, glycoproteins, and basement membrane), each serving distinct roles in signaling and structural functions (Chute et al., 2019).

The myocardium contains three distinct types of collagen fibers (Johnson et al., 2016). These include epimysial fibers, which envelop the entire myocardium, providing external support; perimysial fibers, which encircle bundles of cells; and endomysial fibers, which wrap around individual cells, offering mechanical support and guiding cell alignment (Robinson et al., 1988). The basement membrane accounts for 20% of the ECM, while glycoproteins and proteoglycans make up the structural matrix (4% of the cardiac ECM). These components play a pivotal role in facilitating the propagation of action potentials generated by stimulating cells. The complex structural composition of the ECM, combined with the dynamic activities of the cells, leads to highly variable mechanical properties of the myocardium during a single cardiac cycle (Chen et al., 2008).

Moreover, the ECM serves as a reservoir for growth factors, cytokines, chemokines, proteases (e.g., matrix metallopeptidases), protease inhibitors (e.g., tissue inhibitors of metalloproteinases), and non-coding RNAs, such as microRNAs (Jourdan-LeSaux et al., 2010).

In terms of spatial organization, the ECM is divided into two primary regions: the pericellular matrix (which includes the basement membrane) and the interstitial matrix. Collagens I and III are the predominant constituents of both matrices, providing structural and mechanical support to the tissue. The pericellular matrix is composed of a network of molecules, which includes fibronectin, collagen IV, laminin, procollagens, hyaluronic acid (HA), and proteoglycans (Chang et al., 2016) that interact with each cell through cell surface receptors, such as integrins, facilitating cell processes like differentiation and migration (Corda et al., 2000).

The extracellular microenvironment exhibits a cell-modulatory nature due to continuous remodeling of ECM composition and structural rearrangement. Additionally, bioactive peptides known as matrikines result from enzymatic degradation of ECM macromolecules (Maquart et al., 2005). The balance between ECM synthesis and degradation significantly influences cell responses in terms of proliferation, migration, and even cell death.

Fibroblasts play a major role in the production and remodeling of cardiac ECM under both homeostatic and pathological conditions. However, other cell types, including endothelial cells, smooth muscle cells, and CMs, also contribute to ECM synthesis, particularly in the basement membrane (Pelouch et al., 1993).

Interactions between cells and ECM environment are mediated by various transmembrane cell surface receptors, with integrins being a prominent example (Valiente-Alandi et al., 2016). Integrin-mediated adhesions represent the most common and well-understood cell-ECM interactions (Howard and Baudino, 2014).

Integrin receptors have also direct or indirect interactions with actin cytoskeleton filaments. Following activation, these interactions initiate multiple signaling cascades that govern various cellular responses (Askari et al., 2009). Furthermore, the linkage between ECM, integrins, and the cytoskeleton also plays a crucial role in mediating mechanotransduction signaling (Sun et al., 2016).

Understanding the role of the ECM in modulating cell behavior is crucial for developing *in vitro* culture systems capable of replicating *in vivo* microenvironments and for creating systems capable of replacing or regenerating damaged cardiac tissue.

## 3 3D printing methods

The application of cardiac patches as a possible solution to damaged heart tissue was one of the first foci of tissue engineering (Bader and Oberpriller, 1978). Cardiac patches are required to exhibit, at their best, a series of properties, such as processability in different shapes and sizes, mimicry of the mechanical properties of the native tissue, and a geometry that stimulates cardiomyocytes to elongate and align (Ghofrani et al., 2022). Various methods, labeled as conventional, have been explored to produce cardiac patches. These include solvent casting/particulate leaching (SCPL) and electrospinning (ES) among others (Billiet et al., 2012; Ghofrani et al., 2022). SCPL, for example, easily produces polymeric porous scaffolds with predefined 3D shapes by using sacrificial particles of variable dimensions (Sin et al., 2010). ES is a well-known process that can fabricate randomly entwined micro-to-nano polymeric filaments, generating a fibrous “mat” that provides a large surface-area-to-volume ratio (Zhao et al., 2015). These methods have evolved over the years. ES, for example, was upgraded by involving a rotating spinneret and multiple vertical collector bars to tackle some of its limitations and produce highly uniform fibers (rotary-jet spinning) (Rogalski et al., 2017). Nonetheless, conventional methods struggle to reach satisfying products, which may lack in ECM structure mimicry, mechanical properties, reproducibility, pore interconnectivity or control over the final architecture (Sin et al., 2010; Billiet et al., 2012; Zhao et al., 2015; Ghofrani et al., 2022). Given their complexity, detail-oriented control over architectural cues and cell activity are required to produce promising cardiac patches (Bhattacharyya et al., 2021).

Additive manufacturing (AM) has contributed to overcoming some of the challenges faced by tissue engineering. In particular, 3D printing, an AM ramification, is a very versatile technique for fabricating (and easily reproducing) tissue constructs, based on both natural or synthetic biomaterials. This is accomplished by producing scaffolds in a layer-by-layer fashion following the indications extrapolated by 3D models extracted from medical images, such as cardiac magnetic resonance, computer tomography or ultrasound scans, or CAD software, which allow the fine tuning of the scaffold dimensions, porosity and biological-related features. The rationale behind these complex arrangements is to achieve a native ECM - mimicking construct (Khademhosseini and Camci-Unal, 2018; Bhattacharyya et al., 2021; Ghofrani et al., 2022).

A further branch of 3D printing is 3D bioprinting, which is receiving increasing attention as a promising fabrication method for tissue engineering constructs. By incorporating cells in the biomaterial inks used for scaffold production, 3D bioprinting has led to the development of novel bioinks, which allow precise control not only on the deposited material, but also on cells positioning, interactions, ECM production and tissue organization (Roy et al., 2018). The cell types included in bioinks for CTE are mostly cardiomyocytes, endothelial cells, smooth muscle cells and fibroblasts. Mesenchymal stem cells (MSCs) and human induced pluripotent stem cells derived cardiomyocytes (hiPSC-CMs) also represent a valued choice for cardiac patch engineering (Kozaniti et al., 2021; Li et al., 2022). The innovative approach of bioprinting has led to the development of various technologies that allow cell-laden constructs to be manufactured according to a complex macro- and microgeometry and directly placed in a cell culture environment or a patient anatomical site (Kozaniti et al., 2021). The main challenges of designing these bioinks are ensuring their biocompatibility, processability with no harm to cells and the design of appropriate mechanical features (Roy et al., 2018). The fundamental scaling laws for the printing process have been shown in a recent publication (Schubert, 2022).

The advantages offered by 3D printing technologies can also be exploited to overcome the current limitations of 2D cell cultures. As a matter of fact, 3D scaffolds and bioprinted constructs demonstrate exceptional adherence to the biological 3D microenvironment as they are able to reproduce the biochemical, architectural and cell-interaction features and cues that are naturally available during embryonic development (Borovjagin et al., 2017).

**FIGURE 2:**
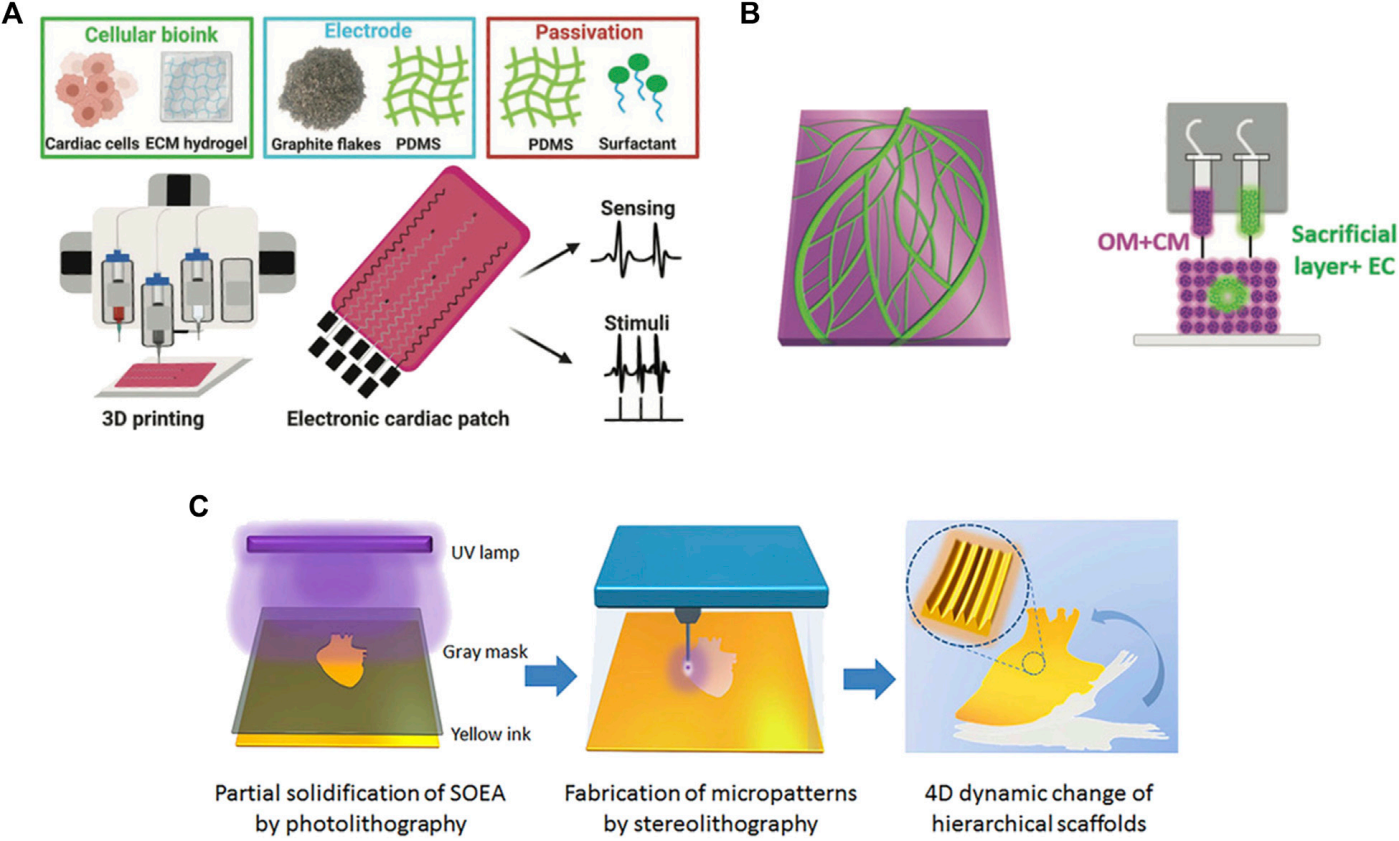
Examples of 3D printing techniques used to produce cardiac patches: **(A)** extrusion based-printing; **(B)** FRESH technique; **(C)** UV-photolithographic technique. Adapted, respectively, from Asulin et al. (2021) (reproduced under the terms of the Creative Commons CC BY license), Noor et al. (2019) (reproduced under the terms of the Creative Commons CC BY license), Miao et al. (2018) (reproduced with permission from Institute of Physics, Great Britain).

Among 3D printing techniques, those being currently addressed as interesting for cardiac patch production include inkjet printing, extrusion printing, freeform reversible embedding printing (FRESH), laser-assisted printing, UV-based printing and melt electrospinning writing (Borovjagin et al., 2017; Cui et al., 2018; Wang et al., 2021b; Kozaniti et al., 2021; Ghofrani et al., 2022) (Figure 2). In Table 1, a summary of the main features of each technique is provided and in Table 2 an overview of 3D printing/bioprinting applications in the field of CTE is presented. In the following sections, the authors will discuss the aspects of these techniques that are relevant for the fabrication of a cardiac patch.

**TABLE 1:** Summary of the main features of currently available 3D printing/bioprinting techniques.

Technology	Production method	Production principle	Features	Limitations	References
*Inkjet*	Drop-on-demand	- Thermal	- High printing resolution (20–100 μm)	- thermal and mechanical stresses	Borovjagin et al. (2017), Jang et al. (2017), Cui et al. (2018), Alonzo et al. (2019), Gardin et al. (2020), Wang et al. (2021b), Kozaniti et al. (2021), Ghofrani et al. (2022)
		- Piezoelectric	- Up to 30 k cells per drop, up to 1,000 drops/s	- inks are not processable	
			- Minor effects on cells	- limited printed cell density	
*Extrusion Based Printing*	Material filament extrusion	- Mechanical (screw-based)	- cell densities close to physiological human CMs values	- Bioink viscosity is a critical parameter	Borovjagin et al. (2017), Jang et al. (2017), Cui et al. (2018), Roy et al. (2018), Alonzo et al. (2019), Wang et al. (2021b), Ghofrani et al. (2022)
		- Pneumatic		- Modest resolution (100 μm)	
				- High shear-stress	
*FRESH*	Extrusion in a support bath	A Bingham-like behaving medium supports the printed contructs	- Addresses the poor mechanical properties exhibited by many interesting hydrogels	- Caution is required for delicate structures or cell-laden hydrogels	Madden et al. (2010), Hinton et al. (2015), Wang et al. (2021b)
*Laser Assisted Printing*	Laser radiation mediates either the extrusion of biomaterials of their crosslinking	- Laser pulse–mediated expulsion of ink from bi-layer metal slide (LIFT)	LIFT	LIFT	Gaebel et al. (2011), Verheugt (2015), Borovjagin et al. (2017), Jang et al. (2017), Roy et al. (2018), Alonzo et al. (2019), Ghofrani et al. (2022)
		- Laser-based crosslinking (MPP)	- high resolution (∼20 μm) and high-speed (ejection frequency up to 5 kHz) printing systems	- not commercially available	
			- cell density up to 10^8^ cells/mL	- High cost (200 k$)	
			- prints any biomaterial with viscosity ranging from 1 to 400 mPa s	- Careful optimization of various parameters (i.e., bioink viscosity, crosslinking/gelation kinetics of the collector and laser parameters) is required	
			- up to a single cell per pulse	MPP	
			- High CMs viability (>90%)	- macro-scale products are difficult to obtain	
			MPP		
			- ability to print (crosslink) soluble and structural ECM proteins		
			- can print details from 3μm to 300 nm		
*UV-based printing*	UV-crosslinking of light sensitive materials	-Beam-scanning (spot by spot)	- can process various biomaterials in a wide range of viscosities (1–2000 mPa s)	- high cost	Lin et al. (2013a), Kim et al. (2014), Borovjagin et al. (2017), Jang et al. (2017), Cui et al. (2018), Izadifar et al. (2018), Roy et al. (2018), Alonzo et al. (2019), Liu et al. (2020), Wang et al. (2021b), Kozaniti et al. (2021), Ghofrani et al. (2022)
		- Mask projection (one whole layer at a time) (DLP)	- accuracy in cell placement and optimised survival	- long printing time (resolvable with DLP)	
			- able to process nanocomposite bioinks or create micro- or nanopatterned surfaces	- difficulty to implement multi-material strategies	
				- the mechanical strength of the printed product may present inhomogeneities	
*Melt Electrospinning Writing*	Voltage powered technique used to produce 3D micro-fibrous scaffolds	Consists of a polymer loaded spinneret, positively charged with respect to a collector plate able to move in the x-y plane	- precisely defined scaffolds	- Voltage, syringe diameter and distance between syringe and plate need to be precisely controlled	Castilho et al. (2018), Olvera et al. (2020), Ghofrani et al. (2022), Ainsworth et al. (2023)
			- can process polymers that are not dissolvable in any solvent		
			- can be used to produce structures with adequate properties for cardiac cells (mechanical anisotropy and electroconductivity)		

**TABLE 2:** Overview of cardiac patches based on natural biomaterials and fabricated by 3D printing/bioprinting.

Technology	Materials	Cells	Functionalization strategy	Ref
Inkjet	Alginate and gelatin	Primary feline adult cardiomyocytes and HL1 cardiac muscle cell line	-	Xu et al. (2009)
	Alginate	Mouse fibroblasts (NIH3T3)	-	Christensen et al. (2015)
Extrusion based	Heart-derived decellularized ECM	Human cardiac progenitor cells (hCPCs)	Vascular endothelial growth factor (VEGF) in the MSC laden bioink to promote neovascularization	Jang et al. (2017)
		Human turbinate tissue-derived mesenchymal stem cells		
	Non-mulberry silk fibroin	Human umbilical vein endothelial cells (HUVECs)–in the bioink	Carbon nanotubes for electrical conductivity	Mehrotra et al. (2021)
	Polyethylene glycol di-methacrylate (PEGDMA)	Neonatal rat cardiomyocytes–seeding on printed structures	Interleukin-10 (IL-10)-loaded GelMA microspheres with immunomodulatory activity and calcium peroxide (oxygen concentration enhancement, cell viability up to 5 days in hypoxic environment)	
	Gelatin methacryloyl (GelMA)			
	Alginate/gelatin	Neonatal mouse cardiac cell spheroids or free (non-spheroid) human coronary artery endothelial cells with fibroblasts	-	Roche et al. (2021)
	Alginate	HUVECs	-	Delkash et al. (2021)
	Egg-white			
	Decellularized pig omentum	Neonatal rat ventricular cardiac cells		Asulin et al. (2021)
	Microbial transglutaminase (mTgase) crosslinked gelatin	Human MSCs	3D printed line features (contact guidance)	Tijore et al. (2018)
		Cardiomyocytes		
	Type I collagen	Reprogrammed human adipogenic mesenchymal stem cells to form purkinje cells	-	Tracy et al. (2020)
	Alginate, GelMA	HUVECs (in the bioink)	-	Zhang et al. (2016)
		Neonatal rat cardiomyocytes		
		hiPSC-CMs		
	Porcine heart decellularized ECM and medical grade COLTRIX (Type-1 atelo-collagen)	Neonatal rat cardiomyocytes	-	Das et al. (2019)
	Genetically engineered multi-domain proteins (ASP - leucine zipper derived and A35m - mussel foot protein derived)	-	Hydrogels loaded with recombinant human follistatin-like 1 (FSTL1) (known to improve cardiac function and survival in mouse and swine models)	Jiang et al. (2022)
	Alginate	Rat ventricular cardiomyocytes and Cardiac fibroblasts	Gold nanorods for improved electrical conductivity	Zhu et al. (2017)
	GelMA			
	Fibrin-based composite hydrogel (fibrinogen, gelatin, aprotinin, glycerol, and hyaluronic acid) as bioink	Neonatal rat ventricular cardiomyocytes	-	Wang et al. (2018)
	Gelatin, glycerol, and hyaluronic acid as support (sacrificial) gel			
	Decellularized ECM from heart, adipose and cartilage tissues	Human adipose-derived stem cells and human inferior turbinate-tissue derived mesenchymal stromal cells	-	Pati et al. (2014)
		Rat myoblast cells (L6, ATCC CRL-1458)		
	Non-mulberry silk fibroin, PEGDMA, GelMA	Human induced pluripotent stem cell derived cardiac spheroids	-	Mehrotra et al. (2020)
	Alginate	HUVECs and hiPSC-CMs	-	Maiullari et al. (2018)
	Polyethylene glycol-Fibrinogen			
	Alginate	Bone marrow mesenchymal stem cells	-	Liu et al. (2021)
	Gelatin			
	Furfuryl−gelatin and fibrinogen solutions	hiPSC-CMs	-	Anil Kumar et al. (2019)
		Human cardiomyocytes (AC16 cell lines)		
	Methacrylated collagen (MeCol)	Human coronary artery endothelial cells	MeCol micropatterning, for topographical stimuli	Izadifar et al. (2018)
	Alginate		Carbon nanotubes incorporation, for conductivity	
	Hyaluronic acid/gelatin	Human cardiac-derived progenitor cells (hCMPCs)	-	Gaetani et al. (2015)
	Double bioink	hiPSC-CMs	-	Chikae et al. (2019)
	-Cells, Thrombin and hyaluronic acid			
	-Fibrinogen and hyaluronic acid			
	Decellularized cardiac ECM and GelMA	Human cardiac progenitor cells	-	Bejleri et al. (2018)
FRESH	Alginate-based hydrogels (RGD modified alginate/Calcium Gluconate/Gelatin/Matrigel for the core; calcium cross-linked alginate/alginate sulfate/extracellular vesicles for the shell)	Human monocytic THP-1 cells	Extracellular vesicle delivery system	Bar et al. (2022)
		GFP expressing human fibroblasts	RGD modified alginate for better cell-matrix interactions	
	Human or pig decellularized omenta (bioinks)	hiPSC-CMs	-	Noor et al. (2019)
	Endothelial cells laden gelatin hydrogel (sacrificial ink)	hiPSC derived endothelial cells		
	Alginate microparticles, xhantan gum supplemented growth medium (FRESH support medium)	Neonatal rat cardiac cells, HUVECs and lumen-supporting fibroblasts		
Laser	Decellularized cardiac ECM	hiPSC-CMs, smooth muscle cells and endothelial cells	-	Gao et al. (2017)
UV-based	GelMA/PEGDA	hiPSC-CMs	4D morphing of the scaffolds driven by light-induced graded pattern of stress (solvent-activated relaxation: swelling-induced stretching and dehydration-induced bending)	Cui et al. (2020)
		Human mesenchymal stem cells		
		Human endothelial cells		
	GelMA or hyaluronic acid glycidyl methacrylate and PEGDA	Neonatal mouse ventricular cardiomyocytes	Micropatterning of the constructs for topographical stimuli	Liu et al. (2020)
	Soybean oil epoxidized acrylate	Human bone marrow mesenchymal stem cells	Micropatterning for topographical stimuli	Miao et al. (2018)
			4D shape changing with immersion in ethanol	

### 3.1 Inkjet printing

Natural polymers, such as alginate and gelatin, have been successfully processed by 3D inkjet for CTE. Various biomaterial parameters are of interest for inkjet printing: biocompatibility, mechanical stability, printing resolution, cost, scalability, and permeability to oxygen/nutrients/waste products. Bioinks, on the other hand, are commonly designed in the form of cell-laden hydrogels or decellularized-ECM (cells encapsulated in natural or synthetic polymers or in a decellularized allogenic or xenogenic tissue). Hydrogels are able to accommodate living cells and possess tuneable mechanical, chemical and degradation properties, ensuring a close resemblance to the native cardiac tissue ECM (Gardin et al., 2020). Decellularized tissues attract attention for their natural content of proteins, proteoglycans and glycoproteins, allowing for the production of biologically and biochemically complex microenvironments (Gardin et al., 2020). While many natural polymer-based hydrogels have been used to encapsulate cells, ECM-based bioinks are costly and may present immunogenicity issues (Borovjagin et al., 2017; Cui et al., 2018; Wang et al., 2021b; Ghofrani et al., 2022).

Several aspects of the 3D inkjet printing process should be further improved. On the one hand, cells and materials are exposed to thermal and mechanical stresses; moreover, the success of inkjet printing relies on the crosslinking of the inviscid bioinks it can process and, if not properly carried out, it can lead to inadequate scaffold mechanical properties. In addition, inkjet may lack in droplet uniformity and directionality, which in turn results in a limited printed cell density, which is instead a fundamental parameter to obtain a functional cardiac patch (Borovjagin et al., 2017; Cui et al., 2018; Alonzo et al., 2019; Wang et al., 2021b).

### 3.2 Extrusion based printing

Extrusion based printing (EBP) is particularly promising in the field of CTE thanks to its affordability, versatility, wide choice of biomaterials and capability of obtaining patient-specific 3D porous scaffolds in a layer-by-layer fashion (Cui et al., 2018; Alonzo et al., 2019; Ghofrani et al., 2022). Obtained scaffolds have been implanted *in vivo*, cultured *in vitro*, or utilised for *ex vivo* applications (Roy et al., 2018). Nonetheless EBP can deposit cell densities close to the physiological human cardiomyocyte values (10^8^–10^9^), making it relevant in CTE (Wang et al., 2021b).

A successful EBP process depends on many parameters, such as nozzle size and geometry, temperature, extrusion force/pressure, post curing method and extruder planar speed. Surface tension is a very important factor as it strongly affects printability in terms of quality, resolution and printed line diameter. Minimising this factor by using highly viscous bioinks, rapidly crosslinking or drying them post printing or printing on a hydrophobic surface can improve the outcome of an EBP process (Cui et al., 2018; Ghofrani et al., 2022). Bioink viscosity is another critical parameter. Several studies in literature show that printability and tolerable cell stress relies on viscosity values between 30 and 6 × 10^7^ mPa s (Cui et al., 2018).

Nevertheless, EBP entails a series of limitations, for example, the difficulty of choosing a printable/compatible material, calibrating the extruder and printing plate to minimise any three-dimensional geometrical mismatch. Moreover, it presents a lower resolution (∼100 μm (Alonzo et al., 2019)), when compared to other fabrication methods, and a high output shear-stress, which may impact cell viability, if the material viscosity is over 6 × 10^7^ mPa s or a thin nozzle is used (Wang et al., 2021b). Shear thinning hydrogels, such as gelatin, represent a possible solution to viscosity and shear stress related problems. In fact, their viscosity decreases with the shear rate, enabling them to protect cells during extrusion and gain structural stability once the shear is released (Wang et al., 2021b).

### 3.3 Freeform reversible embedding printing

The FRESH technique represents a noteworthy advancement due to its substantial enhancement of print precision and its capacity to address geometric intricacies. This precision assumes particular significance within the domain of CTE, where the faithful replication of the complex heart architecture is imperative for the development of effective cardiac patches (Wang et al., 2021b).

FRESH stands out further by facilitating the direct 3D printing of hydrogel inks with pronounced biological relevance, including alginate, fibrin, collagen type I, and Matrigel. These hydrogel materials closely emulate the characteristics of native cardiac tissue, establishing an optimal foundation for the creation of cardiac patches that can seamlessly integrate with the recipient heart. The adaptability of FRESH extends to its adeptness in managing multifaceted multi-material constructs and nonplanar geometries, a capability supported by its utilization of dual syringe-based extruders. This feature empowers researchers to explore various material combinations and formulate intricate designs that were previously beyond the reach of traditional AM techniques (Hinton et al., 2015).

Among the most compelling demonstration of FRESH potentialities, there is its utilization of medical imaging data to produce complex and mechanically robust biological structures. This showcases its exceptional precision and faithfulness in reproducing the intricate architecture of the heart. Furthermore, FRESH is capable of fabricating perfusable structures based on MRI data, such as the right coronary artery vascular tree. These structures encompass internal lumens and bifurcations characterized by sub-millimeter wall thicknesses (Hinton et al., 2015; Lee et al., 2019).

Ultimately, FRESH accessibility emerges as a salient advantage. While other 3D printing methodologies often require substantial equipment expenses and specialized expertise, FRESH is founded on open-source hardware and software. Furthermore, the employment of a cost-effective gelatin slurry, processable with readily available consumer blenders, makes FRESH accessible to a broader spectrum of researchers and institutions (Hinton et al., 2015).

### 3.4 Laser-assisted printing

Laser-assisted printing (LAP) is a family of high resolution and high-speed printing systems, which have been demonstrated the ability to produce 2D or 3D tissue constructs with high cellular viability and cell density up to 10^8^ cells/mL (Alonzo et al., 2019; Ghofrani et al., 2022). LAP technologies include laser-induced forward transfer (LIFT), multi-photon polymerisation (MPP) and matrix-assisted pulsed laser evaporation among the others (Ghofrani et al., 2022).

Any biomaterial with viscosity ranging from 1 to 400 mPa s is a suitable candidate for LIFT. LIFT can produce droplets of constant size (down to ∼20 μm (Borovjagin et al., 2017)) and shape, providing control on the number of cells deposited up to a single cell per pulse (up to 5 kHz (Borovjagin et al., 2017)). Velocities of the printed droplets can reach 150 m/s, leading to high-speed procedures that are not harmful to cells (which may be damaged by velocities higher than 200 m/s). Moreover, in the CTE field, cardiac cell viability has been shown to remain above 90%, without compromising cell function or morphology. Lastly, LIFT is capable of multi-material printing sessions, allowing the production of multicellular constructs, characterized by high resolution and functionality (Gaebel et al., 2011; Verheugt, 2015).

However, there are three main downsides to LIFT: it is not commercially available, which heightens its cost, ranging around 200,000 US$ per unit; the high resolution comes at a cost, as it depends on a series of parameters that need to be carefully optimised, such as bioink viscosity, layer thickness of the biomaterial side, crosslinking/gelation kinetics of the collector and laser parameters; ribbon preparation is costly, time-consuming and challenging, especially if it is designed to be composed of multiple cell types (Borovjagin et al., 2017; Alonzo et al., 2019).

Multiphoton excitation is a LAP technique, which focally crosslinks a solution layer by layer. The innovativeness of this approach resides in its ability to print (crosslink) soluble and structural ECM proteins (collagen, laminin, fibronectin and others) to form complex 3D structures (matrices or fibres) at sub-micron level resolution and with high (85%) fidelity to the CAD model. While this means that macro-scale products are difficult to obtain given the limited printing speed and fidelity that could be obtained, the detail that can be printed with this process (300 nm to 3 μm) is hardly vincible (Borovjagin et al., 2017).

### 3.5 UV-based printing (stereolitography)

Stereolithography (SL) shows promising features for cardiac patch bioprinting, such as its ability to process bioinks and to bioprint high cellular densities. The interest stems from its accuracy in cell placement and optimised survival, given the absence of a nozzle and thus the related shear stress. Interestingly, SL is also able to process nanocomposite bioinks or create micro- or nanopatterned surfaces and volumes, which in turn can lead to improved cardiac cell proliferation, attachment and elongation (Kim et al., 2014; Izadifar et al., 2018; Alonzo et al., 2019; Ghofrani et al., 2022). When dealing with bioinks, the printing parameters and photo-initiators must be optimised to preserve cell viability, phenotype and function from the possible UV or free radical mediated damage, unless visible light is used as the photo-curing source (Borovjagin et al., 2017; Alonzo et al., 2019). SL presents a series of limitations, such as its high cost (Kozaniti et al., 2021), long printing time and difficulty to implement multi-material strategies (Alonzo et al., 2019). Moreover, the mechanical strength of the printed product may present inhomogeneities and repeated laser exposure may result in undesired 3D structures (Borovjagin et al., 2017). Cell sedimentation within the photocurable hydrogels is another issue with SL, which leads to inhomogeneous cell distributions/mechanical properties. This obstacle has been addressed by modifying the bioresin to match the density of cells to the hydrogel density (Lin et al., 2013a; Wang et al., 2021b). Concerning the time consumed, DLP noticeably speeds up the process, bypassing the need for x-y (planar) motion. Each layer is derived from a digital model adequately sliced and is reproduced with high resolution (50–100 μm). DLP is also able to produce complex cell-laden scaffolds. This has been verified for murine cardiomyocytes, making DLP a valid candidate for CTE (Liu et al., 2020).

### 3.6 Melt electrospinning writing

Melt electrospinning writing (MEW) is an interesting processing method for CTE: obtained scaffolds can enhance cell attachment, proliferation, infiltration and alignment, as well as vascularisation. Moreover, MEW can be used to produce structures with adequate properties for cardiac cells, such as mechanical anisotropy and electroconductive characteristics (Castilho et al., 2018; Olvera et al., 2020; Ghofrani et al., 2022).

A recent study by Ainsworth et al., 2023 combined both MEW and EBP with the aim to fabricate a thick, functional cardiac patch with a precisely patterned pre-vascular pathway (Ainsworth et al., 2023). The combination of the two technologies, in which a hexagonal MEW is printed first, followed by dual extrusion-based bioprinting step used for the printing of a prevascular pathway in the shape of a left anterior descending artery within a myocardial construct has a number of advantages. On the one hand, shape fidelity of soft hydrogels can be improved, on the other hand mechanically weaker hydrogels can be processed when reinforced with mechanically robust MEW fibers, while preserving the cellular composition, type and density.

## 4 Natural biomaterial-based bioinks

One of the main challenges in the 3D printing/bioprinting process is ink development. The ideal bioink should in fact satisfy several ideal requirements, which include: high biocompatibility, ability to hold living cells; mechanical stability after printing; printability with high resolution; permeability to oxygen, nutrients and metabolic wastes; and immunocompatibility. Additionally, it should be low cost and enable industrial scalability (Chung et al., 2013; Gopinathan and Noh, 2018).

Usually, bioinks for CTE are made of hydrogels, and cells are encapsulated into the bioinks or seeded on printed constructs to promote cardiac tissue growth. Natural polymers are currently attracting significant attention as hydrogel-based bioinks. This interest stems from their inherent biocompatibility and bioactivity, which ensure the desired behavior of cells and minimize inflammatory responses when implanted in the patient body. The characteristics of the chosen bioink play a pivotal role in influencing cell viability, proliferation, and morphology following the printing process (Malda et al., 2013). The bioink must possess printability, meaning it should be well-suited for creating a stable 3D construct. The key challenge in bioink design lies in enhancing printability while simultaneously preserving cell viability (van Kampen et al., 2019).

When selecting a bioink the basic characteristics to consider are mainly viscosity, gelation and the crosslinking capacity (Malda et al., 2013). Differences between the produced construct and the initial design are influenced by bioink characteristics (Kačarević et al., 2018). Indeed, the mechanical properties of bioinks, including characteristics such as elastic modulus and rheological features, are of fundamental importance (Schrüfer et al., 2020). In general, bioinks should enable consistent and precise deposition, followed by rapid and non-toxic solidification. These factors significantly influence resolution, as well as cell viability, during and after printing (Highley et al., 2015; Chimene et al., 2016; Ouyang et al., 2016).

The most popular hydrogels for 3D printing/bioprinting are alginate and collagen. Other highly used hydrogels are fibrin, gelatin and other natural materials of both protein and polysaccharide nature (Stapenhorst et al., 2021). In the next sections, an overview of the main natural materials used in combination with 3D printing/bioprinting for CTE applications will be provided.

### 4.1 Proteins

#### 4.1.1 Collagen

Collagen has been widely used for cardiac patch fabrication, exploiting the ability of this natural material to provide a matrix that resembles the natural ECM (Knott and Bailey, 1998). Over 80% of the cardiac ECM is formed by type I collagen while type III collagen represents 11% of the cardiac ECM in early adulthood. Collagen enables cell adhesion and differentiation and thus it is widely applied as cell substrate for cardiac applications (Dai et al., 2009; Araña et al., 2014).

There are various examples related to the use of collagen as a bioink for heart tissue regeneration (Izadifar et al., 2018; Das et al., 2019; Lee et al., 2019; Tracy et al., 2020). For example, Lee et al. reported the 3D printing of collagen using FRESH approach. By controlling the pH-driven gelation process, they achieved a filament resolution of 20 µm and created a porous microstructure that facilitates cellular colonization and microvascularization. In addition, the method enabled the fabrication of human heart components of adequate mechanical strength and multiple dimensions, from capillaries to a whole organ. For example, cardiac ventricles were 3D printed incorporating human cardiomyocytes, which showed the desired synchronized contractions (Lee et al., 2019).

#### 4.1.2 Gelatin

Gelatin and its derivatives, attract immense attention as hydrogel-based bioinks. This is primarily due to its cost-effective production, as well as favorable biocompatibility. Additionally, its transparent structure is of paramount importance for facilitating cell monitoring. Furthermore, it presents photocrosslinking capabilities and offers the advantage of adjustability in terms of physical and chemical properties (Ying et al., 2018; Xiao et al., 2019). Moreover, its mechanical features can be tuned by various types of crosslinking strategies (chemical modification with glutaraldehyde or genipin, photo-crosslinking, etc. (Hoque et al., 2015; Naahidi et al., 2017)). On another note, gelatin possesses intrinsic functional groups, which can be available both for cross-linking and for functionalization with biomolecules. Among these, cell adhesion cues make gelatin an ideal platform for cell growth (Roy et al., 2018). All these features demonstrate the similarity between gelatin and natural ECM, and demonstrate that gelatin represents a valuable and highly sought hydrogel for CTE (Djagny et al., 2001; Lapomarda et al., 2021).

**FIGURE 3:**
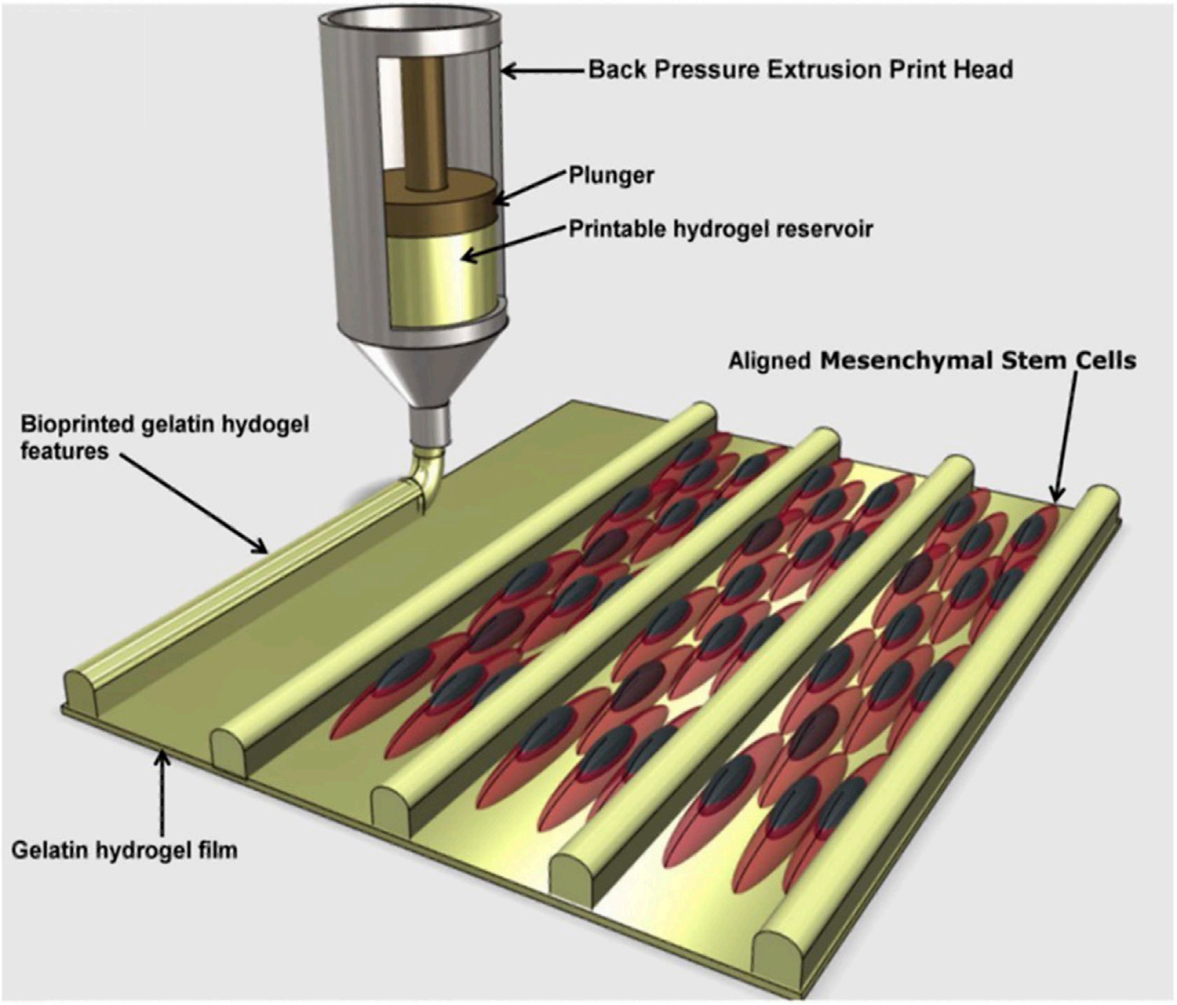
A diagram depicting the production of cell-aligning features (contact guidance) on a gelatin hydrogel film. The printed microchannels favoured human mesenchymal stem cells (hMSCs) alignment and differentiation in myocardial lineage, as well as contractile functionality of cardiomyocytes. Adapted from Tijore et al. (2018), under the terms of the Creative Commons Attribution 3.0 licence.

Tijore et al., for example, 3D bioprinted microchanneled gelatin hydrogel, crosslinked with the microbial enzyme transglutaminase (Tijore et al., 2018). The microchannels acted as contact guidance features for seeded human mesenchymal stem cells (hMSCs) and native CMs, promoting hMSCs commitment to myocardial lineage differentiation and CMs contractile functionality (Figure 3). The final construct displayed aligned and elongated cells, anisotropic properties and synchronized beating (Tijore et al., 2018).

Gelatin was also used as a sacrificial ink by Noor et al. to encapsulate patient-derived endothelial cells and create mathematically optimized vascular ducts in a 3D printed patient-specific cardiac patch. The patch exhibited optimal blood vessel architecture, while completely matching the immunological, anatomical and biochemical properties of the cell donor (Noor et al., 2019). Finally, gelatin is a fundamental ingredient for FRESH applications, which are based on the usage of a gelatin microsphere printing bath. Gelatin microspheres were observed to inherently imprint the extruded hydrogels with a microporous structure, leading to enhanced interactive properties (Hinton et al., 2015; Bar et al., 2022).

#### 4.1.3 Silk fibroin

Silk fibroin (SF) is another natural biomaterials which shows several desirable qualities for tissue engineering applications. In particular, the nonmulberry SF (n-SF) derived from the silk glands of the endemic Indian silkworm variety *Antheraea assamensis* shows excellent potential as a material for CTE (Patra et al., 2012; Mehrotra et al., 2017). In addition to the well-known binding sequence arginine-glycine-aspartic acid (RGD), SF contains other cell-binding motifs, particularly poly-arginine. These motifs have the capacity to interact effectively with integrin isoforms found in cardiomyocytes, leading to accelerated cardiomyocyte attachment and growth (Patra et al., 2012; Mehrotra et al., 2017). In addition, this protein has another interesting feature that concerns the ratio of the amorphous regions to β-crystallites in its secondary structure (Mehrotra et al., 2019), which is responsible for a relatively high elastic modulus and tensile properties (Qi et al., 2017; Guo et al., 2018). Based on these properties, n-SF protein can be exploited in bioinks to improve their mechanical strength while keeping superior biological properties, especially for CTE.

A nSF based biomaterial ink was developed (Mehrotra et al., 2020) for engineering the myocardial tissue. The ink incorporated n-SF, in a hydrogel based on polyethylene glycol dimethacrylate (PEGDMA), and GelMA. The ink was characterized in terms of its physicochemical properties, investigating its ability to fabricate 3D nonvascular anisotropic cardiac constructs and vascularized myocardial tissue. It was observed that the n-SF based ink was suitable to fabricate anisotropic cardiac constructs, which showed mechanical properties, in particular elastic behavior, similar to that of the native heart tissue. Additionally, this ink promoted the functional maturation of cardiomyocytes, maintained their cytoskeletal structure, and enhanced their beating potential. Moreover, an innovative gel embedding-based bioprinting technique was employed to develop a vascularized myocardial tissue. The silk-based ink not only enabled the encapsulation of beating human induced pluripotent stem cell-derived cardiac spheroids (hiPSC-CSs) while preserving their viability, but also facilitated the embedding of HUVECs channels. The endothelial cells, encapsulated within the channel bioink, gradually migrated towards the channel edges, leading to the formation of endothelial-based vasculature. After maturation in culture, the vascularized myocardial tissue expressed proteins characteristic of both cardiomyocytes and HUVECs.

#### 4.1.4 Fibrin

Fibrin is a native biopolymer formed through polymerization of fibrinogen, a soluble glycoprotein present in the blood, triggered by the thrombin enzyme (Scheraga, 2004; Wolberg, 2007). Fibrin has been extensively utilized in the creation of tissue engineering scaffolds due to its advantageous properties, including ease of preparation and handling, biocompatibility, and biodegradability. With reference to 3D bioprinting techniques fibrinogen alone struggles to maintain a stable 3D printed shape, regardless of its concentration, because the viscosity of fibrinogen solution remains constant despite changes in shear (de Melo et al., 2019), which causes the collapse of the printed scaffold. Several approaches have been proposed to overcome this drawback, such as combining fibrinogen with other printable polymers (Rutz et al., 2015; Anil Kumar et al., 2019), employing a support bath to embed the printed scaffold (Hong et al., 2015; de Melo et al., 2019), or crosslinking the hydrogel during printing (de la Vega et al., 2018). Fibrin-based bioinks with tunable printing properties have been developed by smart combination of fibrinogen solution with other printable polymers. Printable hydrogels are those that possess adjustable gelation properties, allowing them to be extruded while retaining their 3D shape fidelity after printing (Kyle et al., 2017). Thermo-reversible biomaterials are considered within this context; they undergo a sol-gel transition, being soluble within a specific temperature range and creating a gel beyond a particular transition point. Gelatin is a commonly used biomaterial in 3D bioprinting, and as a result, combining biomaterials with suboptimal rheological properties, such as fibrinogen, with gelatin can enhance printability for fabricating 3D structures based on fibrin (Xu et al., 2007). An alternative strategy involves 3D bioprinting fibrinogen-based bioinks within a support bath, using layer-by-layer deposition methods. This approach enables rapid cross-linking of the bioink by supplementing the bath with a cross-linking agent (Hinton et al., 2015). This bioprinting method effectively preserves the shape integrity of the printed construct. Hinton *et al.* reported that fibrinogen inks can form complex structures with high precision and reproducibility, when printed in a gelatin support bath (Hinton et al., 2015). Another approach for 3D bioprinting biomaterials that lack a sol-gel transition, like fibrinogen, involves *in situ* cross-linking. In this technique, the bioink and the cross-linking agent are extruded separately from distinct needles, allowing for their individual and simultaneous deposition. Different ratios of the bioink and cross-linking agent can be combined to fabricate heterogeneous solid structures (Dai et al., 2017; Costantini et al., 2018).

3D printed fibrin scaffolds are suitable for developing cardiac tissues exhibiting aligned cells (Wang et al., 2018). For example, Wang *et al.* reported a biofabrication method based on extrusion to deposit neonatal rat ventricular cardiomyocytes using a fibrin-based bioink (Wang et al., 2018). Obtained results indicated the development of cardiac tissue composed of consistently aligned, densely populated, and electromechanically connected cardiac cells, which could spontaneously and synchronously contract in culture.

In another study, a photopolymerizable gelatin-based bioink incorporating fibrin was used to fabricate constructs embedded with human induced pluripotent stem cell-derived cardiomyocytes (iPS-CM) or CM cell lines with cardiac fibroblasts (Anil Kumar et al., 2019). The cell-laden bioprinted constructs were cross-linked to maintain a herringbone pattern using a two-step process. The first step was furfuryl-gelatin cross-linking by visible light; then the second step consisted of fibrinogen chemical cross-linking, through the use of thrombin and calcium ions. A highly connected porous structure of the constructs was observed, which enabled high viability and proliferation of the cardiomyocytes printed within the structure, which also expressed the troponin I cardiac marker.

#### 4.1.5 GelMA

Gelatin methacryloyl (GelMA) hydrogel is a bioink that has garnered significant attention in research due to its ease of synthesis, cost-effectiveness, excellent biocompatibility, transparent structure for cell monitoring, photocrosslinking capability, and support for cell viability. Another advantageous property is that the mechanical properties of GelMA can be modulated by adjusting parameters such as concentration, photocrosslinking time and method, degree of functionality, and crosslinking efficiency, in order to meet the requirements of different body tissues, including the cardiac one (Rajabi et al., 2021).

Several studies in literature have investigated GelMA-based bioinks for CTE. For example, a new GelMA-based bioink with a low concentration (4.5% w/v) and low degree of methacrylate (20%) was proposed by Colosi et al. (2016). Neonatal rat cardiomyocytes were seeded on the top of the printed structures to demonstrate their efficacy in CTE. The results obtained showed a desired cell viability in all parts of the scaffold, which also resulted in a good support for the synchronous beating of heart cells.

Vascularization is another key requirement for CTE. Many studies in literature (Chen et al., 2012; Lin et al., 2013b) have shown that GelMA hydrogels can support the formation of vascular network in different printed tissues. *In vitro* investigations have demonstrated that GelMA hydrogel, when combined with human blood-derived and bone marrow-derived mesenchymal stem cells, can facilitate the development of an extensive capillary network. Jia et al. (2016), for example, created a novel bioink utilizing GelMA to enhance the formation of blood capillary networks. The cell viability assessments and live and dead staining of the printed structure confirmed the outstanding cellular response on this material.

#### 4.1.6 Proteinaceous gel

Proteinaceous gels (PGs) can be defined as biomaterials constructed from self-assembling subunits (Jing et al., 2008). These materials are part of a recent trend in material science which aims at copying “natural material designs” with very interesting properties that have been achieved over millions of years of evolution (Fernández-Colino et al., 2014). The biomimicry of these designs allows the reproduction of complex cell-interactions (adhesion, protease sensitivity, cytokine release, the fibrillar and viscoelastic nature of ECM, etc.) (Fernández-Colino et al., 2014). An example of PGs are those based on recombinant proteins, which can be homogenously produced in great quantities from microbial fermentation. These materials exhibit biocompatibility, biodegradability, reduced immunogenicity and their modular design allows for the fabrication of functional-blocks that can be rationally integrated in order to exhibit specific properties (such as stiffness, biological cues, controlled release) (Jiang et al., 2022). These modules can self-assemble to create a hierarchical structure with close similarity to natural ECM, representing an extremely interesting possibility for applications in tissue engineering (Jing et al., 2008; Huang et al., 2014), and are appreciated for their easiness of synthesis (Jing et al., 2008).

**FIGURE 4:**
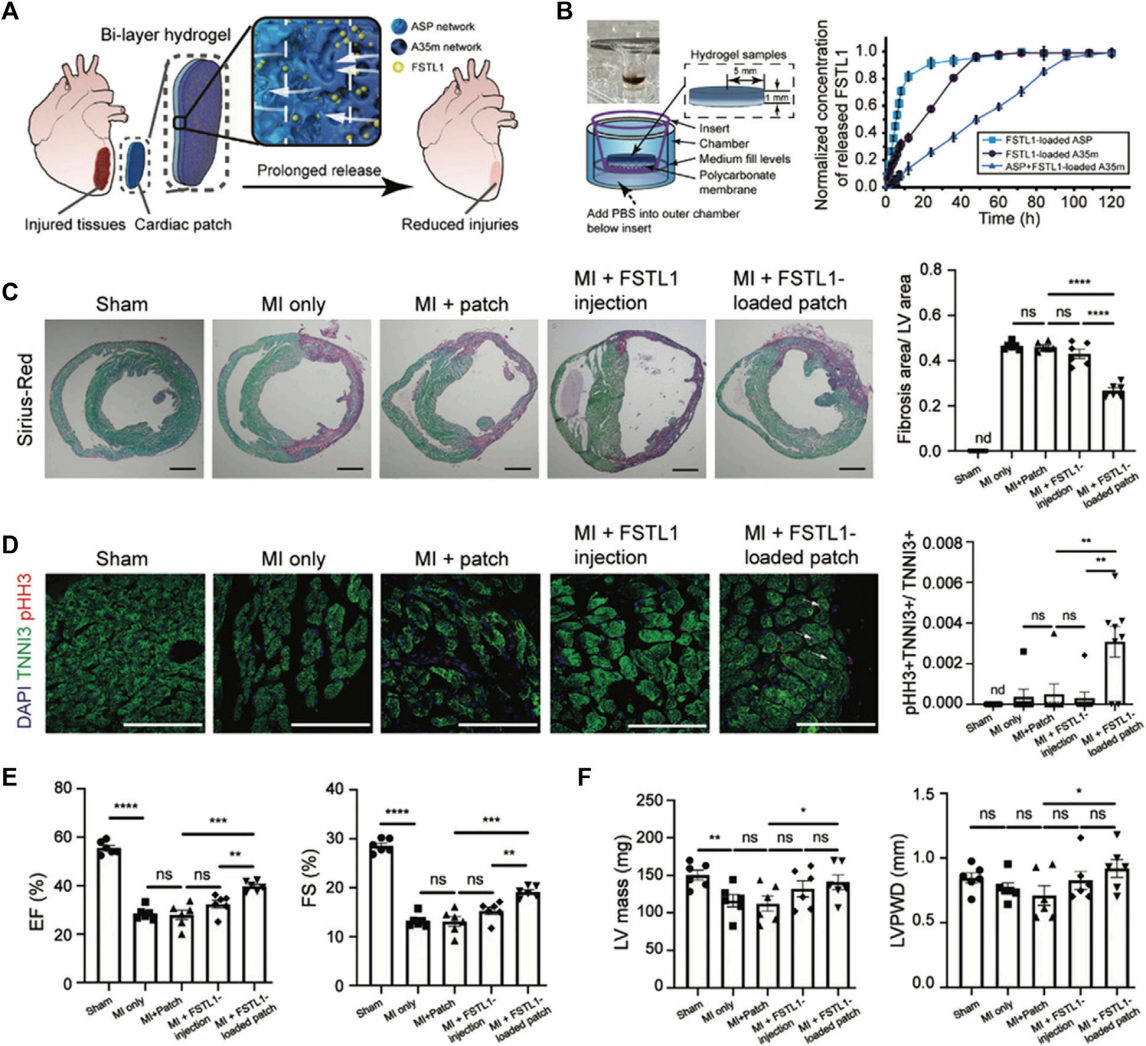
Improvements in heart function in a MI model through treatment with the FSTL1-loaded bi-layer ASP-A35m hydrogel cardiac patch developed by Jiang et al. (2022): breakdown of the experimental design and main results. **(A)** Schematic of the experimental design: human FSTL1 protein (shown as green dots) was loaded into the outer layer (A35m hydrogel, dark blue layer). The FSTL1-loaded outer layer was then combined with the inner hydrogel (ASP hydrogel, light blue layer). The FSTL1-loaded bi-layer ASP-A35m hydrogel cardiac patch was then implanted on the epicardium of a mouse model of acute MI. **(B)** Transwell device used to evaluate FSTL1 release from ASP, A35m, and ASP-A35m hydrogels (on the left); results of FSTL1 releasing tests in PBS (on the right). **(C)** Fibrosis analysis. Representative Sirius-Red stained sections for various groups: MI only, ASP-A35m hydrogel cardiac patch, FSTL1-loaded ASP-A35m hydrogel cardiac patch, FSTL1 injection, and sham (*n* = 6 for each), at 7 days post MI. Scale bars: 1 mm. Histograms on the right show the distribution of fibrosis areas. **(D)** Cardiomyocyte proliferation. Immunofluorescence staining against TNNI3 (green) and pHH3 (red) on hearts from different treatment groups: MI only, ASP-A35m hydrogel cardiac patch, FSTL1-loaded ASP-A35m hydrogel cardiac patch, FSTL1 injection, and sham (*n* = 7 for each). Scale bars: 100 µm. Histograms on the right display proliferating cardiomyocytes (indicated by arrows). **(E)** Left ventricular ejection fraction (EF) and fractional shortening (FS), at 7 days after MI. **(F)** LV mass and LV posterior wall thickness at end-diastole (LVPWD), at 7 days after MI. Reprinted from Jiang et al., 2022. Reproduced with permission from John Wiley and Sons.

Jiang et al. tried to address CTE issues by producing a genetically engineered shear-thinning leucine zipper-based PG composed of two layers, each addressing one aspect of the final scaffold: mechanical strength, interfacial adhesion to cardiac tissue, biocompatibility and biodegradability for the first layer, a physical barrier to resist burst pressure from the beating of the heart and drug release capabilities for the second (Figure 4). The thus-designed cardiac patch displayed significant wound repair capacity in a transmural puncture murine model, improving cardiac functionality (Jiang et al., 2022).

### 4.2 Polysaccharides

#### 4.2.1 Alginate

Alginate has been thoroughly investigated to fabricate 3D scaffolds or injectable hydrogels for TE applications, and its properties have been comprehensively studied (Ozbolat and Yin, 2013; Zhang et al., 2017). The material is in fact appreciated for its biocompatibility, easily tunable mechanical properties, controllable degree of crosslinking, flexibility and low cost (Cattelan et al., 2020). However, alginate is recognized to be biologically inert, as it lacks cell adhesive cues; therefore, adhesive peptides, such as the RGD sequence, have been immobilized on alginate scaffolds to enhance cell interaction for tissue engineering purposes. In the work of Shachar et al., RGD-modified alginate promoted cell adhesion, survival and recovery of cardiomyocytes, allowing a better organization of CM myofibrils and myofibers. Moreover, cells grown on this scaffold showed a similar structure and organization to native cardiac tissue (Shachar et al., 2011).

With specific reference to the application of 3D printing for CTE, alginate was demonstrated to favor the differentiation, proliferation and printing of human cardiac derived progenitor cells (Gaetani et al., 2012; Alonzo et al., 2019). Gaetani et al. have designed an alginate-based scaffold with homogeneously seeded hCMPCs, which maintained high viability (∼90%) enhancing the expression of typical genes associated with early cardiac transcription factors (Gaetani et al., 2012). Alginate was also used as a FRESH support medium for CTE applications by Noor et al. (2019).

### 4.3 Protein/polysaccharides blends

In addition to proteins or polysaccharides alone, blends of proteins and polysaccharides, synergically combining the complementary advantages offered by the starting components, are receiving growing interest as scaffold materials for CTE. Such blends can also be particularly advantageous, as they can resume the protein/polysaccharide nature of native cardiac ECM (Rosellini et al., 2018b). Alginate is the most investigated polysaccharide because of its mild crosslinking conditions. It has been used in combination with gelatin (Xu et al., 2009; Liu et al., 2021; Roche et al., 2021), GelMA (Zhang et al., 2016; Zhu et al., 2017), collagen (Izadifar et al., 2018), fibrinogen (Maiullari et al., 2018), egg white (Delkash et al., 2021) or a protein mix (Bar et al., 2022).

In terms of alginate/gelatin bioinks, different weight ratios of the two polymers were investigated, ranging between 96/4 (Xu et al., 2009), to 62/38 (Liu et al., 2021) and up to 33/67 (Roche et al., 2021), respectively. The two polymers can be dissolved in PBS (Xu et al., 2009) or NaCl (Liu et al., 2021) and then sterilized using an autoclave after complete dissolution (Xu et al., 2009) or, alternatively, the powders can first be sterilized under UV light and then dissolved in complete culture medium (Roche et al., 2021). The aim of the alginate/gelatin blend was to combine the possibility to perform ionic cross-linking on the cellular patch (given by alginate) and the capability to improve cell adhesion due to the presence of integrin-binding motifs (given by gelatin) (Roche et al., 2021). The versatility of alginate/gelatin bioink has been firmly established. The incorporation of gelatin provides control over the rheological properties, thereby enhancing printability, all while preserving the durability characteristics during culture. Additionally, this bioink allows for cardiomyocyte contractility and the formation of endothelial cell networks (Mancha Sánchez et al., 2020).

When alginate is combined with GelMA, GelMa is used to provide bioactivity and matrix metalloproteinase-sensitive groups, while alginate is used to avoid the physical cross-linking of GelMA chains, maintaining the bioink viscosity over time at room temperature (Zhu et al., 2017). Moreover, this mixture enables a dual step cross-linking procedure. First, during the bioprinting process, the ionic cross-linking of the alginate component is obtained by exposing the bioink to a CaCl_2_ solution; then, after printing, a stable gelation is achieved by UV crosslinking of the GelMA component (Zhang et al., 2016).

Alginate has also been investigated in combination with collagen (Izadifar et al., 2018). Collagen was chosen as it promotes excellent cell response, in terms of adhesion, differentiation and mobility. It is difficult to bioprint, due to its poor self-supporting property and low viscosity, but this drawback was compensated by the excellent printability of alginate, which in turns suffers of poor cellular adhesion.

In a different study conducted by Maiullari et al., a bioink composed of alginate and modified fibrinogen was extruded using a custom microfluidic printing head. This specialized printing head was designed with an outlet channel connected to a co-axial needle system that acted as the extruder. This setup allowed for the rapid solidification of the printed hydrogel fibers (Maiullari et al., 2018). During the bioprinting process, these two biopolymers played distinct roles: alginate served as a temporary material template, facilitating the controlled hydrogel deposition; meanwhile, fibrinogen created a matrix capable of supporting cell spreading and differentiation during extended *in vitro* cultures. The simultaneous co-extrusion of the bioink and the cross-linking solution resulted in the immediate formation of hydrogel fibers at the tip of the inner needle, allowing for the precise assembly of high-resolution scaffolds. Additional stability was conferred through UV cross-linking of the modified fibrinogen. Before *in vitro* maturation, a significant amount of alginate was removed from the 3D printed structures through washing with a potent chelating agent. This step was essential because alginate, due to its lack of adhesion molecules and limited biodegradation *in vivo*, is not an ideal matrix for culturing induced pluripotent stem cells (iPSCs).

More recently, an innovative combination of alginate with eggwhite was investigated (Delkash et al., 2021). Chicken egg white serves as an accessible and cost-effective source of albumin, a protein with crucial biological functions, such as protecting and supporting embryo development (Kaipparettu et al., 2008). Moreover, the amino acids released during albumin biodegradation can provide the required nutrients for cells (Fleischer et al., 2014). However, 3D printing of eggwhite is challenging, due to its flow behavior (Godoi et al., 2016). Eggwhite printability can be improved by the addition of NaOH (Chang et al., 2019), however, its use can pose challenges in tissue bioprinting, as it can have adverse effects on cell viability and functionality, including issues with proliferation, morphology, and cytoskeletal distribution (Noriega and Subramanian, 2011). Therefore, in this work an innovative printable bioink was developed, combining the enhanced biological properties of eggwhite with improved bioink printability, provided by alginate (Delkash et al., 2021). The amount of alginate in the blend was chosen to obtain adequate rheological properties for printing patch constructs. Subsequently, the printed patches underwent both mechanical and biological characterization. The findings indicated an elastic modulus that closely resembled that of natural heart tissue. Furthermore, vascular endothelial cells displayed very high viability rates, exceeding 90%, 1 week after the printing process.

In 2022, Bar et al. proposed a 3D bioprinted cardiac patch composed of two layers: a cell-laden inner core and an extracellular vesicle-laden hydrogel as the outer shell (Bar et al., 2022). Appropriate bioink formulations were investigated for both compartments. In particular, for the inner core, and RGD-modified alginate was chosen, to promote the desired cell-matrix interactions as a result of the presence of biochemical cues provided by the adhesive peptide. Nonetheless, ensuring the stability of the construct is another critical consideration when fabricating a cardiac patch, as it is essential for the patch to remain in place over a beating heart. To enhance the bioink viscosity and, consequently, improve mechanical stability after printing, it was decided to incorporate either gelatin alone or a combination of gelatin and Matrigel into the alginate-based bioink solution. Printing parameters, in terms of temperature, pressure and printing velocity, were optimized for the different solutions. Moreover, their effect on laden cells was investigated. Overall, the combination of RGD-modified alginate with gelatin exhibited the most promising potential to promote cell survival and organization.

**FIGURE 5:**
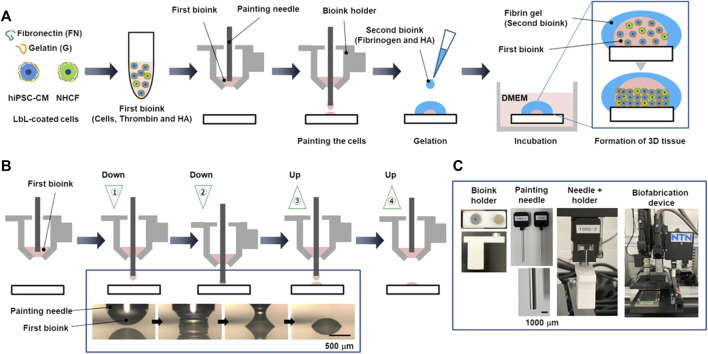
The process of layer-by-layer (LbL) coating of cells and the construction of a 3D heart using a painting needle method, developed by Chikae et al. (2019). **(A)** Schematic illustration of the process, comprising painting of a first bioink (cells, thrombin and hyaluronic acid), subsequent manual addition of a second bioink (fibrinogen and hyaluronic acid) and incubation to form a 3D tissue. **(B)** Details on painting process of the first bioink: the bioink adhered on the needle and it was transferred to the substrate by moving the needle up and down. **(C)** Pictures of painting needle and device. Reprinted from Chikae et al. (2019). Reproduced with permission from John Wiley and Sons.

As an alternative to alginate, hyaluronic acid has been investigated, in combination with gelatin (Gaetani et al., 2015), fibrin (Chikae et al., 2019) or a protein mix (gelatin and fibrin) (Wang et al., 2018). While for Gaetani et al. the aim of the mixture between hyaluronic acid and gelatin was to obtain a good combination of printability and enhanced cell attachment and survival (Gaetani et al., 2015), Chickae et al. developed a two component bioink, in order to construct a 3D cardiac tissue with high cell density, using a custom microscopic painting needle device (Figure 5) (Chikae et al., 2019). In summary, a two-step process was employed. Initially, a high-viscosity bioink containing a dense cell population, thrombin, and hyaluronic acid was applied to a substrate using a painting needle. The bioink adhered to the needle and was then transferred to the substrate with high resolution through up-and-down movements of the painting needle. Subsequently, a second bioink containing fibrinogen and hyaluronic acid was manually added. Upon contact between the gel initiator (thrombin) and gel agent (fibrinogen), a dome-shaped gel structure formed, releasing hyaluronic acid outside the dome during culture. Consequently, the viscosity of the solution inside the dome gradually decreased, causing the cells to settle and form a high-density tissue (Chikae et al., 2019).

### 4.4 Decellularized ECM

It is evident that single natural or synthetic materials are not able to recapitulate the complexity of the native ECM. The ECM of each tissue has distinctive composition and structure, which arise from interactions between the resident cells and their surrounding microenvironment (Frantz et al., 2010). In the process of tissue regeneration, the ideal solution would be to provide cells with a natural microenvironment similar to that of the original tissue.

Decellularized ECM (dECM) could represent the best choice to do this and for this reason it is currently one of the most investigated biomaterials for CTE application. In particular, dECM with stem cells has been used as bioink for 3D bioprinting of functional cardiac construct. dECM offers several advantages, such as biocompatibility, good printability, mechanical strength and no immunogenicity. Moreover, high cell viability, excellent cell adhesion and maturation and easy synchronization with grafted tissue were observed (Qasim et al., 2019).

Pati et al., in 2014, developed a bioprinting method, which involves the use of a novel bioink based on dECM in order to produce cell-loaded structures able to provide an optimized microenvironment conducive to the growth of 3D structured tissues (Pati et al., 2014). Different varieties of dECM-based bioinks, including cardiac dECM, have been employed to showcase the adaptability and effectiveness of bioprinting techniques. A notable advantage of this approach is the utilization of tissue-specific ECM, which offers valuable insights into aspects such as cell engraftment, viability, and sustained functionality over extended periods.

Another interesting example of a dECM-based cardiac patch, generated through bioprinting, involved a dECM bioink laden with human neonatal c-kit positive cells referred to as human cardiac progenitor cells (hCPCs), whose printability was favoured by the inclusion of 5% w/v GelMA (Bejleri et al., 2018). The developed cardiac patch was intended to treat pediatric heart failure in patients with congenital heart defects. The hCPC-laden GelMA-dECM bioinks showed homogeneous distribution of cells and matrix. The cells loaded into GelMA-dECM patches exhibited high viability and robust proliferation for up to 7 days. These cells also displayed enhanced differentiation and angiogenic potential compared to pure GelMA patches, suggesting improved reparative capabilities. The incorporation of dECM resulted in patches with a mechanical modulus similar to that of native myocardial tissue. Furthermore, *in vitro* testing demonstrated that the patches maintained their structural integrity over a period of 21 days. This approach also offers the possibility of customizing patches to match the specific tissue requirements of individual patients. The concept of a patient-specific, 3D-printed patch holds significant translational value. In practice, this could involve the use of autologous hCPCs derived directly from pediatric patients aged 1 week or less, who are undergoing heart surgeries for congenital heart diseases. By utilizing commercially available biomaterials like porcine dECM, hCPC expansion can be followed by rapid and tailored patch manufacturing through 3D bioprinting. This method allows for the inclusion of other commercially available cell sources, such as mesenchymal stem cells, to create a personalized patch that can be directly applied to the patient during surgery.

**FIGURE 6:**
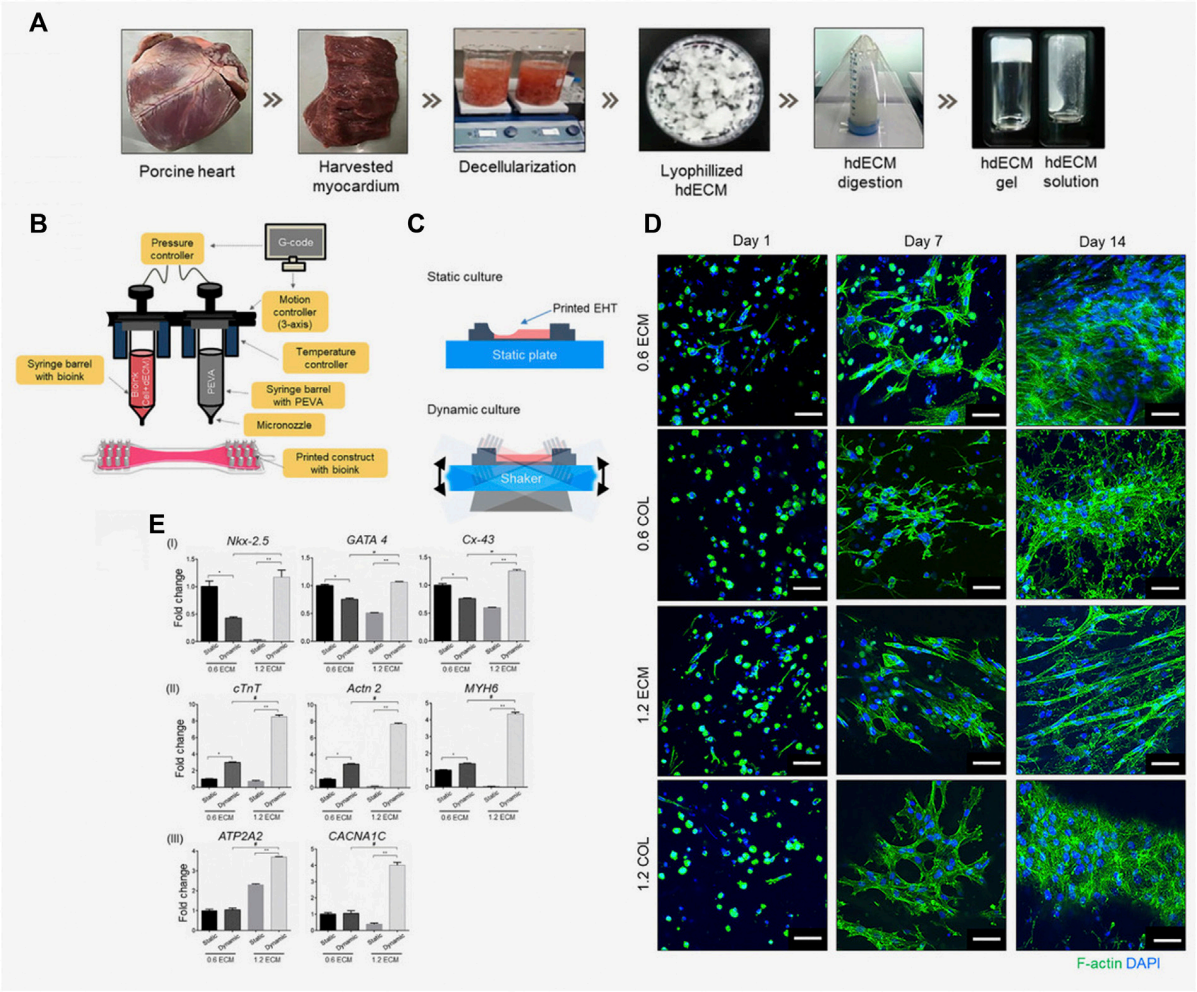
Engineered heart tissue (EHT) fabricated by Das et al. (2019). **(A)** Preparation of the heart tissue-derived decellularized ECM (hdECM) as bioink; **(B)** Fabrication of the EHT using a 3D bioprinter; **(C)** A schematic representation illustrating the culture of printed EHT under both static and dynamic conditions; **(D)** Using fluorescence microscopy, actin filaments formed by the cardiomyocytes encapsulated in the EHT were observed. Images were acquired at different time points (1, 7, and 14 days) for EHTs fabricated using different concentrations (0.6% and 1.2%) of ECM and collagen (COL), used as control. Over time, cardiomyocytes progressively developed more extensive networks of cytoplasmic actin filaments. Scale bar: 50 µm. **(E)** The maturation of cardiomyocytes and their phenotypic changes were analyzed by conducting gene expression analysis using RT-PCR on day 14. Different bioink concentrations (0.6 and 1.2 ECM) and culture conditions (static/dynamic) were compared, and the results are reported with error bars representing the standard deviation. Statistical significance is indicated by *, **, and # for *p*-values lower than 0.05. Adapted from Das et al., 2019. Reproduced with permission from Elsevier.

In 2019, Das et al. reported an enhanced maturation of cardiomyocytes in dECM as compared to pure collagen under similar culture conditions (Figure 6) (Das et al., 2019). More precisely, the 3D-printed engineered heart tissue created with a low concentration of dECM facilitated the early differentiation of cardiomyocytes. The findings from this study suggest that an optimal balance of signals from the ECM-based bioink and the transport of nutrients within the culture environment plays a pivotal role in promoting enhanced cell differentiation and maturation. It becomes evident that the matrix microenvironment and the dynamic conditions of the culture are critical factors that influence interactions between cells and between cells and the matrix. These interactions, in turn, impact the structural organization of cardiomyocytes, subsequently influencing their gene expression patterns and related signaling pathways.

## 5 Strategies for functionalization of 3D printed constructs

Despite significant advancements made in the last years, engineering a fully functional 3D printed cardiac construct to be used as replacement tissue or *in vitro* model is still a challenge, as many unforeseen hurdles must be overcome to achieve the ambitious goal of mimicking the complex physiologic microenvironment. Body tissues are composed of three fundamental components: i) cells, ii) the ECM, which serves as the “natural scaffold” of native tissues, and iii) bioactive molecules and signals that regulate cell proliferation and differentiation. The ECM not only acts as a physical support for cells but also creates a natural environment that fosters cell proliferation, differentiation, and morphogenesis. Consequently, it plays a vital role in tissue regeneration and organogenesis. In situations involving large tissue defects, natural regeneration and repair solely through cell supplementation are challenging, as the ECM is also lost. Therefore, one approach to stimulate tissue regeneration at the site of damage is to artificially construct an environment for cells by providing an ECM-like scaffold. This scaffold facilitates initial cell attachment and subsequent processes of cell proliferation and differentiation, ultimately promoting tissue regeneration (Tabata, 2000; Tessmar and Göpferich, 2007). Given these considerations, it becomes evident that a scaffold must offer more than just the ability to withstand mechanical forces or possess appropriate degradation kinetics. Amongst other properties, the scaffold should recruit desirable cells, favor cell adhesion, promote cell proliferation and guide cell differentiation. Therefore, in the case of 3D printed cardiac patches, in addition to appropriate bioinks and scaffold architectural design, additional scaffold functionalization strategies should be developed to recapitulate all the features of the complex native microenvironment, which are critical regulators of cell fate (e.g., migration, adhesion, proliferation and differentiation) and determine the obtainment of a functional cardiac patch. An overview of the different functionalization strategies developed in combination with 3D printed cardiac patches will be provided in the next sections.

### 5.1 Loading of biochemical signals

The native ECM is a reservoir of bioactive molecules. Therefore, ECM-like scaffolds should provide the means to deliver biochemical signals to cells, to support tissue development. Directly injecting biomolecules in solution into the target site for regeneration is often ineffective, due to the rapid diffusion of the bioactive factor from the injection site, leading to enzymatic digestion or deactivation. To enable the efficient biological function of the bioactive factor, a drug delivery system is necessary (Tabata, 2005). Ideally, the release of bioactive factors should occur gradually over an extended period, reducing the necessity for repeated applications of the factor. Additionally, a localized release of bioactive factors confines their activity to a specific area near the site of the defect, thereby minimizing potential side effects. Incorporating the bioactive factor into a release carrier may also protect it from proteolysis, ensuring prolonged activity retention *in vivo* (Tessmar and Göpferich, 2007). Several strategies for loading bioactive agents into a scaffold have been developed (Tabata, 2005), including the direct loading of the biomolecule of interest into the polymeric matrix of the scaffold, the coating of prefabricated scaffolds, the covalent binding to the scaffold surface and the loading of the bioactive molecule inside a carrier, which is then included in the scaffold.

The decoration of scaffolds with bioactive molecules is a functionalization strategy that has also been investigated for a 3D printed cardiac patch. Among the biomolecules used in cardiac patch development there are growth factors, protein-based biomolecules secreted by cells, which are able to stimulate specific cellular responses, such as growth, proliferation and differentiation, thus promoting myocardial healing and repair at the infarct site (Tallawi et al., 2015). Among protein-based biomolecules used in CTE there are stromal-derived growth factor (SDF-1) (Hiasa et al., 2004; Rosellini et al., 2021b), insulin-like growth factor (IGF-1) (Musarò et al., 2004; Frati et al., 2020; Rosellini et al., 2021a), and recombinant human follistatin-like 1 (FSTL1) (Ogura et al., 2012; Wei et al., 2015; Jiang et al., 2022).

In 2022, Jiang et al. developed a 3D printed bi-layer proteinaceous hydrogel patch, for heart failure treatment (Jiang et al., 2022). The two layers of the cardiac patch were created using two distinct genetically engineered shear-thinning protein hydrogels based on leucine zippers, specifically ASP and A35m. The outer layer was constructed using the A35m hydrogel, known for its denser internal porous structure and stronger cohesion compared to ASP. This characteristic allows it to effectively extend the storage and release of therapeutic molecules. In this particular study, the A35m layer was loaded with FSTL1, a molecule known to enhance cardiac function and survival in both mouse and swine models (Ogura et al., 2012; Wei et al., 2015). The therapeutic effectiveness *in vivo* was assessed by implanting the bi-layer cardiac patch loaded with FSTL1 in a mouse model of MI. The results demonstrated that the patch’s ability for prolonged storage and sustained release of FSTL1 led to enhanced proliferation of cardiomyocytes, reduced cardiac fibrosis, and improved cardiac pumping function. This was reflected in increased left ventricular ejection fraction and left ventricular fractional shortening.

Among paracrine factors that play a key role in heart repair, extracellular vesicles (EVs) need to be mentioned (EL Andaloussi et al., 2013). EVs are miniscule vesicles, with a diameter of 30–150 nm and, on the basis of the surface proteins, can be distinguished in apoptotic bodies, microvesicles and exosomes (György et al., 2011). Exosomes, in particular, have been studied extensively. They are phospholipid bilayer-enclosed vesicles, secreted by stem cells, and they contain bioactive molecules (proteins, mRNA and miRNA) inside (Théry et al., 2002). As a result, these molecules play a crucial role in facilitating cell-to-cell communication, orchestrating a range of intercellular processes, and possessing the capacity to decrease inflammation, apoptosis, and fibrosis while promoting angiogenesis (He et al., 2020). Recent studies have demonstrated that EVs, rather than parent stem cells, contribute to treating cardiovascular diseases (Loyer et al., 2014).

**FIGURE 7:**
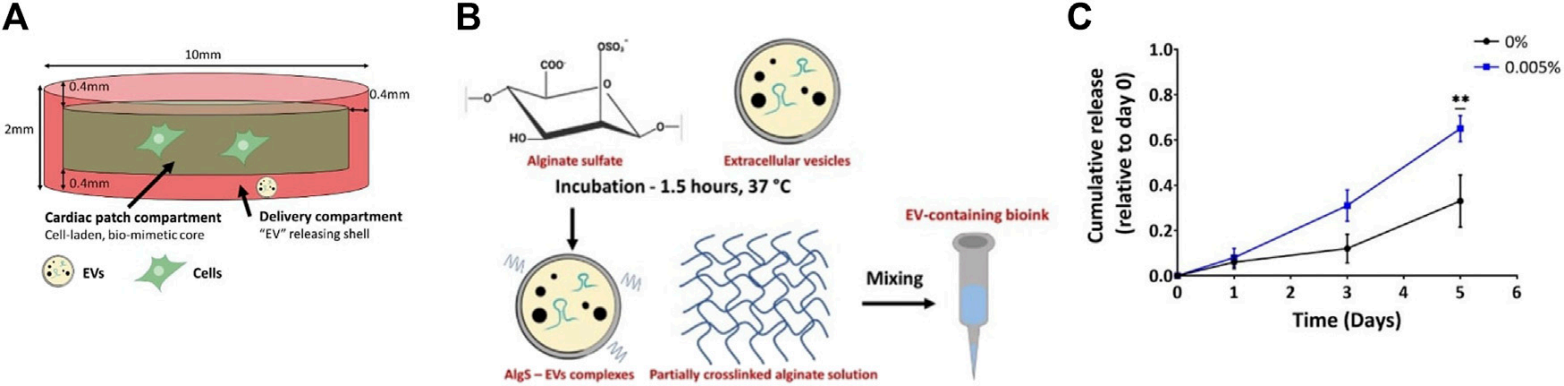
Cardiac patch developed by Bar et al. (2022). **(A)** The designed cardiac patch includes two compartments: an inner core containing cardiac cells and RGD-modified alginate; an outer shell made by alginate and AlgS-EV complexes, for controlled release of EV; **(B)** Scheme describing the preparation of the bioink, containing AlgS-EVs complexes; **(C)** The addition of AlgS (0.005%) is found to prolong the release of EVs from 3D-bioprinted alginate constructs, with respect to alginate alone (0%). Each data point represents the mean ± standard error (*n* = 3, 4) cumulative release. Statistical significance was determined through a two-tailed Student’s t-test at each time point and it is indicated by * for *p* < 0.05 and ** for *p* < 0.01. Adapted from Bar et al., 2022. Open access article distributed under the terms and conditions of the Creative Commons Attribution (CC BY) license.

In 2022, Bar et al. developed a three-dimensional bioprinted cardiac patch, for sustained delivery of EVs (Figure 7) (Bar et al., 2022). The patch was composed of an inner core, containing cardiac cells, and an outer shell, containing EVs, and was aimed to promote cardiac regeneration and improve integration with the surrounding tissue. Alginate-based bioinks were developed for both layers. In particular, for the outer shell, alginate sulfate (AlgS) was used, having affinity binding to EVs. Release tests were carried out, comparing the cumulative release from 3D printed pristine alginate constructs, with that from AlgS. The use of AlgS slightly reduced the percentage of released EVs and prolonged the release. *In vivo* tests in a cardiac injury model need to be carried out to further evaluate the efficacy of this approach.

### 5.2 Immunomodulation

Immunomodulation is an innovative strategy recently introduced to improve the efficacy of currently unsuccessful regenerative approaches (Bloise et al., 2020). This technique aims to modulate the activity of immune cells in conditions of immune hyperactivity or hypoactivity. To achieve this, immunomodulatory agents are used that, by acting on effector cells and on those which inhibit antibody production, are able to determine the direction, amplitude and persistence of immune system cell responses (Singh and Peppas, 2014). In general, multiple immunologic reactions occur during a tissue injury and therefore the immune system is strongly involved in the tissue repair and regeneration process. Immunomodulation is particularly relevant in post-infarction myocardial regeneration, because by modulating the immune response it is possible to strongly influence the overall outcome, as well as the speed of the healing process of the damaged tissue. In MI, cardiomyocyte necrosis stimulates the activation of the adaptive and innate immune system (Nakkala et al., 2021). The resulting inflammatory response is called “sterile inflammation” and is mainly constituted by the infiltration of neutrophils and macrophages (Mokarram and Bellamkonda, 2014).

Macrophages play a pivotal role in both the inflammatory and anti-inflammatory aspects of the healing process, thereby influencing the initial sequence of cellular events following an injury. They typically arrive at the site of injury within 48–96 h after the traumatic event. Their functions include the removal of dead cells and debris, the release of pro-inflammatory cytokines, the secretion of oxygen radicals, and the subsequent production of anti-inflammatory factors to mitigate inflammation. Additionally, macrophages stimulate processes like angiogenesis (the formation of new blood vessels), fibroblast proliferation (important for tissue repair), and fibroblast migration, all of which are essential for effective tissue regeneration. Tissue macrophages originate from circulating peripheral blood monocytes that migrate into tissues in response to various signals. Different macrophage phenotypes can be distinguished, and during the later stages of the tissue repair process, depending on their interactions with other cells and their microenvironment, they can adopt either an M1 or M2 phenotype. The M1 phenotype has a pro-inflammatory function, characterized by the production of inflammatory cytokines such as IL-1β, IL-6, IL-12, IL-23, and TNF-α. In contrast, the M2 phenotype possesses anti-inflammatory properties and is activated by signals like IL-4, IL-13, IL-10, leading to the production of high levels of IL-10, which is an anti-inflammatory cytokine (Mokarram and Bellamkonda, 2014).

From what has been said, the importance of these cells in the resolution of inflammation following MI and in the subsequent tissue regeneration can be deduced. Immunomodulation combined with CTE aims to control the inflammatory environment of the infarcted myocardium, in order to promote tissue regeneration (Ferrini et al., 2019). Indeed, the inflammatory response follows a time-dependent process that progresses through three distinct yet overlapping phases: inflammation, proliferation, and resolution (Farache Trajano and Smart, 2021). Immunomodulation aims to intervene in a timely manner during these processes, trying to promote the survival of cardiomyocytes, the attenuation of the acute inflammatory response and the polarization of macrophages to an anti-inflammatory and reparative phenotype.

In recent years, extensive research has been conducted to develop biomaterials capable of modulating these immunological reactions and assisting in the process of cardiac tissue regeneration (Alvarez et al., 2016; Julier et al., 2017).

**FIGURE 8:**
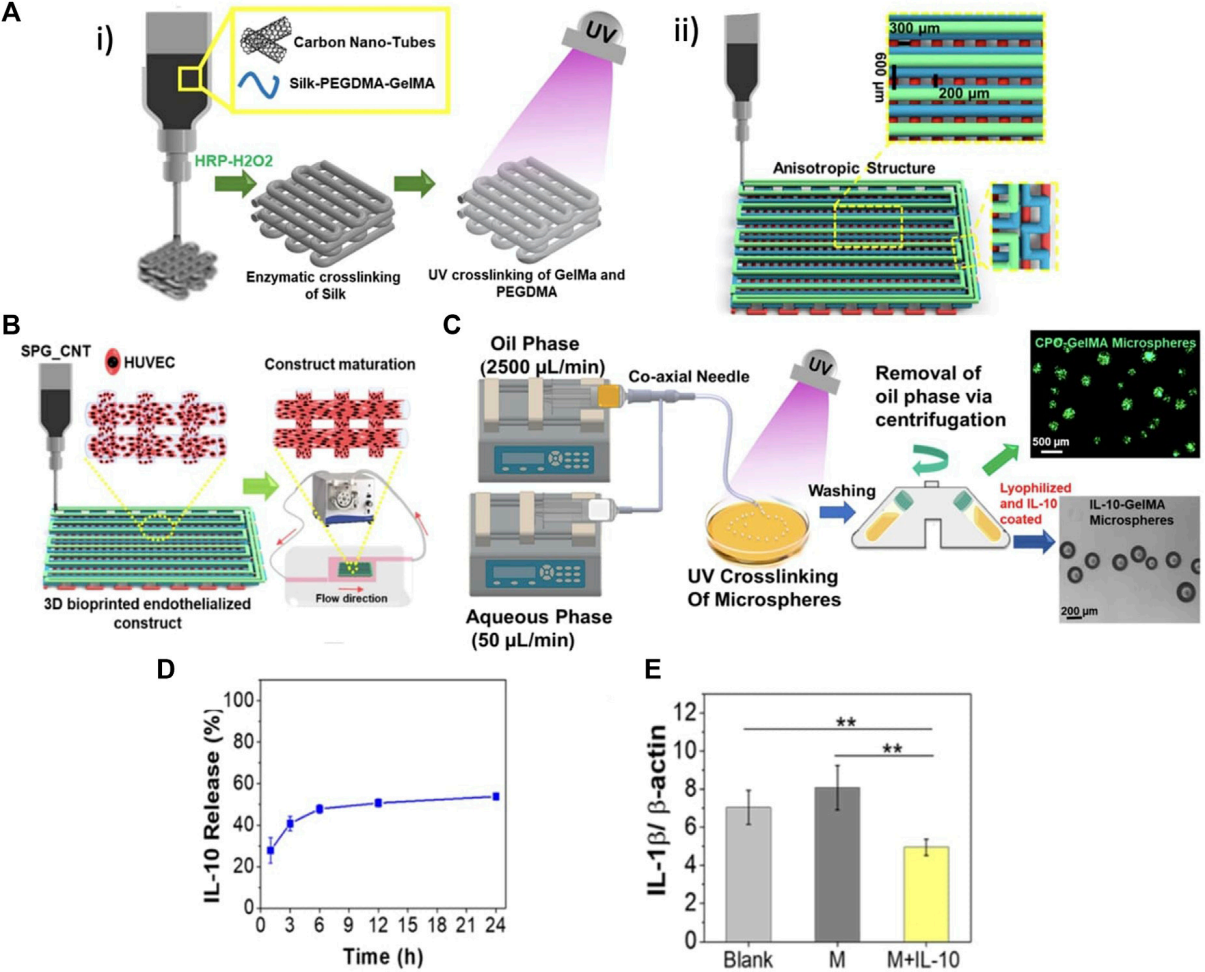
3D bioprinted cardiac patch with immunomodulatory potential, developed by Mehrotra et al. (2021). **(A)** Design of the 3D bioprinted construct: i) a diagram that illustrates the dual cross-linking process carried out on the bioink; ii) a schematic that depicts the design of an anisotropic 3D bioprinted microfibrous scaffold, aiming to fabricate a cardiac construct resembling the native heart tissue. **(B)** Schematic describing the inclusion of endothelial cells and construct maturation within a perfusion bioreactor. **(C)** Fabrication of microspheres loaded with IL-10, using a customized dual syringe-pump coaxial needle system. **(D)** Kinetic of IL-10 release from microspheres. **(E)** Evaluation of the immunomodulatory effects exerted by IL-10-GelMA microspheres on human macrophages, with a focus on decreased secretion of IL-1β in IL-10-GelMA treated group, suggesting an inhibition of pro-inflammatory M1 macrophage polarization. The results are presented as the mean (*n* = 3) with ** indicating *p* ≤ 0.01 and * indicating *p* ≤ 0.5. Adapted with permission from Mehrotra et al. Copyright 2021, the American Chemical Society.

In 2021, Mehrotra et al. developed an immunomodulatory scaffold by 3D bioprinting, using a silk-based conductive bioink with embedded carbon nanotubes (CNTs) (Figure 8) (Mehrotra et al., 2021). In this study, HUVECs were encapsulated within the bioink to create a vascularized construct. These constructs were cultured in a perfusion bioreactor and later seeded with neonatal rat cardiomyocytes. To enhance the acceptance of the 3D bioprinted construct in the harsh ischemic environment following MI, GelMA-based microspheres loaded with calcium peroxide (CPO) and interleukin-10 (IL-10) were integrated into the scaffold. The hypothesis was that oxygen released from CPO microspheres would help maintain cell viability in the hypoxic microenvironment, while the release of IL-10 could promote the polarization of macrophages to the M2 phenotype, facilitating the regeneration process. *In vitro* experiments were conducted to assess the scaffolds’ immunomodulatory capacity by measuring the release of IL-10. The results showed lower expression of M1 phenotype surface markers, reduced levels of pro-inflammatory cytokines (such as IL-1β), and polarization of macrophages towards the M2 phenotype. To evaluate *in vivo* immunocompatibility, the constructs were implanted subcutaneously in rats. Obtained results included integration of the implant with host tissue, a mild immune response, and neovascularization. Overall, the findings suggested that IL-10 microspheres could induce the polarization of macrophages from M1 to M2 phenotype, thus promoting tissue repair. Future studies involving implantation at the infarcted region in a rat MI model will be needed to further verify the efficacy of these scaffolds.

### 5.3 Inclusion of additives for conductivity and electrical coupling

The myocardium possesses distinctive electromechanical properties; it is an electroactive tissue that undergoes spontaneous contractions in response to electrical signal propagation. The electrical coupling between individual cardiomyocytes is among the most crucial factors that facilitate their spontaneous beating function. Ensuring proper electrical coupling is essential for generating contractile forces and enhancing tissue maturation (Kensah et al., 2013). Hence, achieving conductivity is a critical requirement for CTE scaffolds to mimic the electrical conductivity of the native myocardium. Numerous studies in the literature have showcased that the utilization of electroconductive cardiac patches, incorporating materials such as graphene derivatives, carbon nanotubes (CNTs), or gold nanorods (GNRs) (Martinelli et al., 2012; Martinelli et al., 2013; Shin et al., 2014; Jakus et al., 2015), can favour electrical coupling and consequently the development of a functional tissue. At the same time, electroactive scaffolds could also be a more suitable platform for *in vitro* drug testing, by providing biomimicry of native myocardium.

In 2017, Zhu et al. developed GNRs-incorporated GelMA and GelMA/alginate bioinks for 3D printing of cardiac patches, with the aim to improve scaffold conductivity and consequently the electrical coupling with adjacent cardiac cells (Zhu et al., 2017). The printability of gold nanocomposite bioinks was investigated using different GNR concentrations. Using an optimized concentration of GNR, it was possible to extrude cells in nanocomposite bioinks, with a high cell viability (>70%). After bioprinting, cardiac cells resulted homogeneously encapsulated in the constructs. Nanocomposite bioinks exhibited favorable characteristics, including robust cell spreading and proliferation, high metabolic activity, and the development of a uniform and interconnected tissue layer. Additionally, bioinks containing GNRs demonstrated enhanced electrical coupling and improved contractile behavior when compared to bioinks without such additives.

**FIGURE 9:**
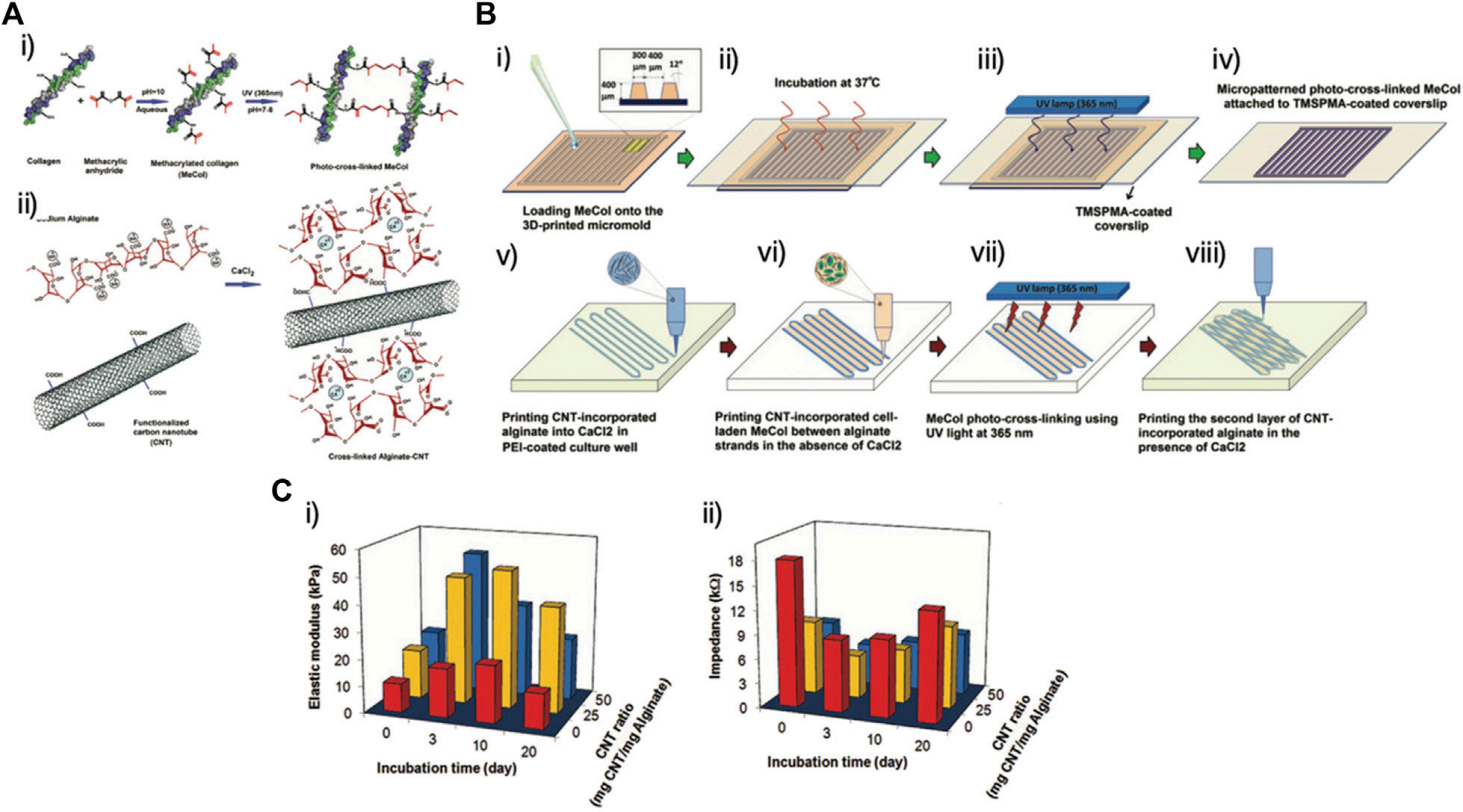
**(A)** Schematic diagram illustrating: (i) the production of MeCol and its cross-linking by UV-light; (ii) alginate cross-linking and creation of covalent bonds between carboxylated carbon nanotubes (CNTs) and alginate, induced by calcium ions. **(B)** The steps involved in the fabrication process, highlighting the micropatterning of MeCol and the bioprinting of the hybrid implant. **(C)** Analysis of variations in the (i) compressive elastic modulus and (ii) impedance (at 5 Hz) of the hybrid 3D-printed construct, in relation to incubation time and CNT mass ratio. Adapted with permission from Izadifar et al., 2018. The publisher for this copyrighted material is Mary Ann Liebert, Inc. publishers.

In a different study, surface-functionalized CNTs were integrated into a combination of alginate and methacrylated type I collagen (MeCol) to facilitate the bioprinting of a cardiac patch. This approach aimed to enhance the stiffness, electrical conductivity, and cellular response of the patch (Figure 9) (Izadifar et al., 2018). Human coronary artery endothelial cells (HCAECs) were incorporated into the patch. Specifically, a UV-assisted 3D bioprinting technique was employed to create a hybrid cardiac patch, comprising a framework of alginate with functionalized CNTs and HCAECs-laden MeCol. The study demonstrated that the inclusion of functionalized CNTs in alginate and MeCol components enhanced the electrical conductivity and mechanical properties of the hydrogels. This improvement was attributed to the formation of a filamentous structure induced by CNTs within the hydrogel matrix. Notably, the creation of a nanofibrous interconnected network, consisting of nanofibers ranging in size from 25 to 500 nm in alginate-coated CNTs, not only enhanced electrical and mechanical properties but also promoted HCAEC attachment and elongation, compared to pristine alginate. Additionally, the hybrid implant exhibited highly favorable biological behavior *in vitro*, including robust HCAEC proliferation, migration, and differentiation, as indicated by lumen-like structure formation.

### 5.4 Promotion of neovascularization

The myocardium is a densely vascularized tissue, and to create physiologically functional tissues, advanced vascularization strategies are necessary. These strategies may involve the incorporation of cells or bioactive factors, such as vascular endothelial growth factor (VEGF) (Calvillo et al., 2003), which has the potential to expedite the vascularization of the engineered construct and, in turn, enhance the survival of the tissue being developed (Tallawi et al., 2015). Nonetheless, when geometric control is lacking, vascular cells tend to form randomly distributed capillary-like structures that result in very slow blood flow, providing limited functional improvement. The direct printing of vascular networks within engineered tissues can therefore represent a promising strategy to promote an efficient vascularization, which favour tissue survival and function and allow the connection to the patient’s vasculature.

**FIGURE 10:**
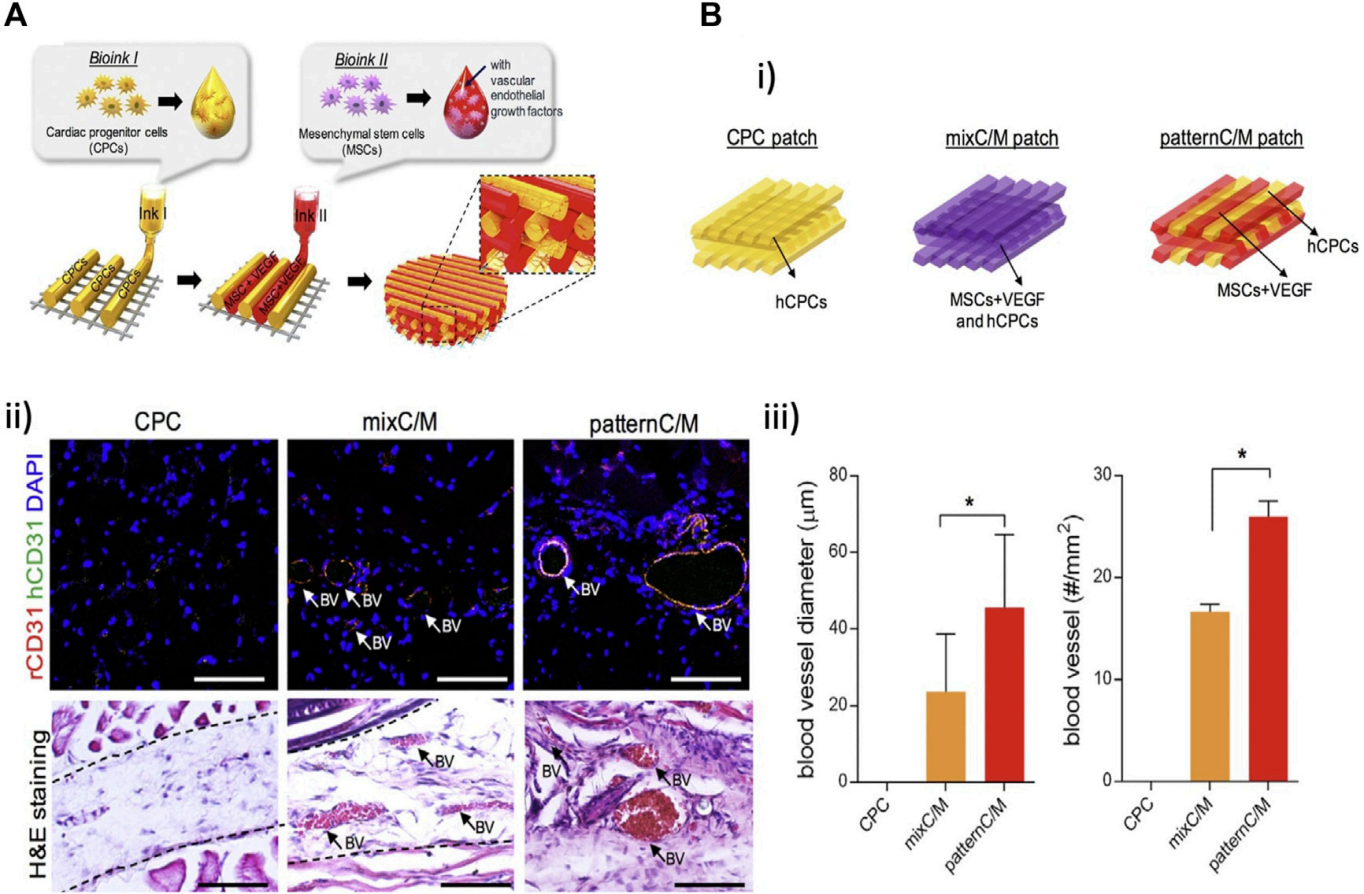
**(A)** Fabrication of a pre-vascularized stem cell patch: two different cell-laden bioinks were alternatively printed on a PCL layer, which provided mechanical suppor. **(B)** The impact of the pre-vascularized stem cell patch on *in vivo* vascular formation: (i) diagrams outlining the different experimental groups; (ii) histological analysis and results of immunofluorescence staining 4 weeks after implantation, with blood vessels (BV) indicated. (iii) Quantitative analysis of blood vessel diameter and number. The error bars represent the standard error of the mean, with statistical significance indicated by (*p < 0.05). Adapted from Jang et al., 2017. Reprinted with permission from Elsevier.

For example, Jang et al. developed a 3D printed pre-vascularized patch, by using as bioink dECM, in combination with human c-kit + cardiac progenitor cells (hCPCs) isolated from human infant-derived heart tissues, mesenchymal stem cells (MSCs), and VEGF, which was chosen to promote a rapid vascularization (Figure 10) (Jang et al., 2017). In particular, three bioinks with different composition were compared: dECM and CPCs (bioink I); dECM, VEGF and MSCs (bioink II); dECM, VEGF, CPCs and MSCs (bioink III). An *in vitro* vascularization test was carried out by culturing structures based on bioink II for 5 days in endothelial cell growth medium. It was observed that VEGF promoted vascular formation, with a morphology very similar to that of vessels formed by microvascular endothelial cells. *In vivo* tests in mice were also carried out to investigate neovascularization and tissue formation. Three different experimental groups were compared, combining dECM with: i) CPCs only; ii) randomly mixed CPCs and MSCs with VEGF (mix C/M); iii) patterned CPCs and MSCs with VEGF alternatively (pattern C/M). After 4 weeks implantation at subcutaneous site, both samples containing VEGF promoted vascularization much better than that with CPCs only. In particular, capillaries were observed in the mix C/M sample, while more vessels with larger dimensions were found in the pattern C/M sample. *In vivo* experiments were conducted using a rat MI model to assess the therapeutic effectiveness of the engineered patch. The pattern C/M patch demonstrated improved cardiac functions, decreased cardiac hypertrophy and fibrosis, enhanced migration from the patch to the infarcted area, as well as the formation of new muscle tissue and capillaries, leading to overall improvements in cardiac function.

### 5.5 Environmental-stimuli responsiveness

4-dimensional (4D) printing is an advanced additive manufacturing process that seeks to create 3D printed structures capable of self-transformation in shape or function in response to environmental stimuli like osmotic pressure, heat, electric current, ultraviolet light, or other energy sources. While 4D printing is still in its early stages of development, it shows significant potential for applications in tissue engineering, including CTE.

Miao et al. used smart natural lipids (soybean oil epoxidized acrylate, SOEA) as bioink to fabricate a 4D dynamic shape-changing cardiac patch (Miao et al., 2018). The photolithographic-stereolithographic-tandem strategy was utilized to create complex micropatterns. The 4D effect refers to the autonomous bending of these structures into rolling shapes, triggered by an external stimulus applied after printing (such as immersion in ethanol for 10 min). This bending behavior likely results from a cross-link density gradient formed during photolithography, enhancing the internal strength of the printed structure. The degree of bending could be controlled by adjusting the scaffold thickness, offering a straightforward means of matching the curvature of damaged tissue, which is crucial for achieving better tissue integration. *In vitro* biological tests, conducted with human mesenchymal stem cells (hMSCs), showed cell alignment and differentiation toward a cardiomyogenic lineage, demonstrating the promising potential of the 4D-fabricated cardiac patch for heart regeneration.

In 2020, Cui et al. developed a 4D cardiac patch with physiological adaptability using beam-scanning stereolithography. They employed a gelatin-based bioink, which consisted of GelMA and PEGDA. This innovative approach aimed to create a cardiac patch that could adapt to physiological conditions, potentially improving its functionality and integration when implanted in the heart (Cui et al., 2020). The research described the fabrication of anisotropic cardiac patches capable of replicating the fiber orientation of the native myocardium. These patches were highly stretchable, allowing them to switch between different fiber arrangements in accordance with the diastole and systole phases of the cardiac cycle. Moreover, they demonstrated a self-morphing capacity, transforming from a 3D flat pattern to a 4D curved architecture in response to solvent-induced relaxation and internal stress. This self-morphing ability enabled the patches to closely match the surface curvature of the heart, improving their biomechanical properties and dynamic integration with the beating heart. The patches were cultured with human induced pluripotent stem cells, human mesenchymal stem cells, and human endothelial cells under physiologically relevant mechanical stimulation. *In vitro* testing revealed enhanced vascularization and cardiomyocyte maturation. Additionally, the patches were tested *in vivo* using a murine chronic MI model, demonstrating increased cell engraftment and vascular supply, indicating their potential therapeutic efficacy for heart repair.

### 5.6 Shape memory effect

Despite notable advancements in the development of functional cardiac patches, their implantation still necessitates an invasive surgical procedure involving an open-chest approach. This factor adds complexity to the procedure and imposes constraints on their therapeutic potential within the field of CTE. The possibility of delivery of a functional cardiac patch via injection would represent a further advancement, reducing the invasiveness of the surgical procedure for implantation. Injectable scaffolds for cardiac repair can be produced by different strategies, such as *in situ* gelation, microgel suspension and shear-thinning gels (Rosellini et al., 2018a). Nonetheless, in all these methods, the shape takes form after the injection process, making it challenging to precisely control the final patch shape and size. Consequently, this may result in irregular shapes, non-uniform macrostructures, and variations in functionality. A cardiac patch combining a preformed shape and injectability could overcome these limitations (Bencherif et al., 2012). Shape memory materials could be helpful for the production of cardiac patches that are implantable through a minimally invasive procedure, as they can resume specific and pre-defined shapes after injection.

In recent years, several studies have emerged in the literature that explore the integration of 3D printing technology with synthetic shape memory materials for the creation of injectable cardiac patches (Locke et al., 2016; Montgomery et al., 2017; Feng et al., 2021). In another research, an injectable and conductive cardiac patch with shape-memory behaviour was produced using natural materials, but through a traditional fabrication technique (Wang et al., 2021a). To the best of our knowledge, a study combining shape memory materials of natural origin and 3D printing for cardiac tissue regeneration is not yet available in literature.

## 6 Applications

### 6.1 Cardiac tissue regeneration

In recent years, 3D printing and 3D bioprinting have found large-scale application in the cardiovascular field. One of the main investigated applications is the production of a patch for repairing the infarcted myocardium. Due to the limited regenerative capacity of cardiomyocytes, the cardiac ECM is gradually replaced by scar tissue as heart failure progresses following a MI. This scar tissue lacks the ability to contract synchronously with the surrounding healthy tissue (Gaetani et al., 2012). In order to compensate for this loss of contractile tissue and ensure an adequate cardiac output, a left ventricular remodeling occurs, which is a pathological process characterized by left ventricular dilatation and altered chamber geometry.

Therefore, the primary goal of tissue-engineered cardiac patches is to stimulate the regeneration of the infarcted myocardium, preventing the formation of scar tissue and facilitating the development of functional tissue that integrates with the heart electrical and mechanical activity. Given the intricate nature of cardiac tissue, 3D printing and bioprinting have emerged as cutting-edge techniques for creating biomimetic cardiac patches capable of replicating the properties of native myocardium. These advanced fabrication methods address a significant challenge in traditional regenerative medicine, which involves engineering complex multicellular structures like cardiac tissue with integrated vascular networks. Bioprinting offers precise control over the placement of biomaterials, live cells, and bioactive molecules, enabling the accurate recreation of the complex composition found in native tissue.

Although the application of 3D printing/bioprinting in the field of CTE is quite recent (the first report dates back to 2014 (Pati et al., 2014)), several *in vivo* studies in animal models of MI have been already reported, with very promising results (Gaetani et al., 2015; Gao et al., 2017; Jang et al., 2017; Maiullari et al., 2018; Cui et al., 2020; Mehrotra et al., 2020; Liu et al., 2021; Mehrotra et al., 2021; Jiang et al., 2022).

### 6.2 *In vitro* cardiac tissue models for drug screening and disease detection

Furthermore, beyond its potential for *in vivo* treatment of MI, CTE has significant applications in the development of *in vitro* cardiac tissue models. These models serve as valuable tools for advancing our understanding of basic cardiac physiology, studying the pathogenesis of heart diseases, and functioning as high-throughput platforms for drug screening. *In vitro* tissue models are increasingly attractive due to their ability to offer higher accuracy and predictability compared to traditional 2D cell culture models. They also address the limitations associated with conventional animal models, including issues related to interspecies variability, the high cost of experimentation, and the time-intensive nature of studies.

As the myocardium is a highly organized tissue, the realization of a functional and biomimetic *in vitro* cardiac tissue model requires an organized cell alignment; therefore, scaffolds for this application should be designed to guide cardiac cell orientation. When compared with other traditional scaffold fabrication techniques, 3D printing technologies offer higher flexibility to produce patterned geometries. For example, Tijore et al. cultivated human mesenchymal stem cells and neonatal rat cardiomyocytes on bioprinted patterned gelatin scaffolds (Tijore et al., 2018). An improved cell elongation, alignment and differentiation was observed for hMSCs on micropatterned hydrogels, with respect to plain ones (Figure 3). Moreover, micropatterned hydrogels promoted CMs alignment and spontaneous beating. In summary, this research demonstrates that the utilization of 3D bioprinting to create microchanneled hydrogel scaffolds promotes the differentiation of stem cells into myocardial cells and provides a conducive environment for the growth and contractility of CMs. Consequently, this approach holds promise for the development of *in vitro* cardiac model systems, which can be valuable for conducting physiological studies and assessing cardiotoxicity.

Although cardiomyocytes are the main cellular component, the cardiac tissue does not contain a single cell type, but a pool of different cells. Hence, the creation of biomimetic *in vitro* cardiac tissue models necessitates not only patterned scaffolds but also the incorporation of various cell types, including endothelial cells, cardiac fibroblasts, and other stromal cell types. This multifaceted cellular composition enables paracrine signaling interactions among cardiac cells and contributes to the maturation of cardiomyocytes within the model. In this regard, 3D bioprinting can offer the unique possibility to construct multi-cellular microscale structures in a single step process. This further characteristic represents a significant advancement toward the development of a biomimetic *in vitro* platform for basic research and drug discovery.

**FIGURE 11:**
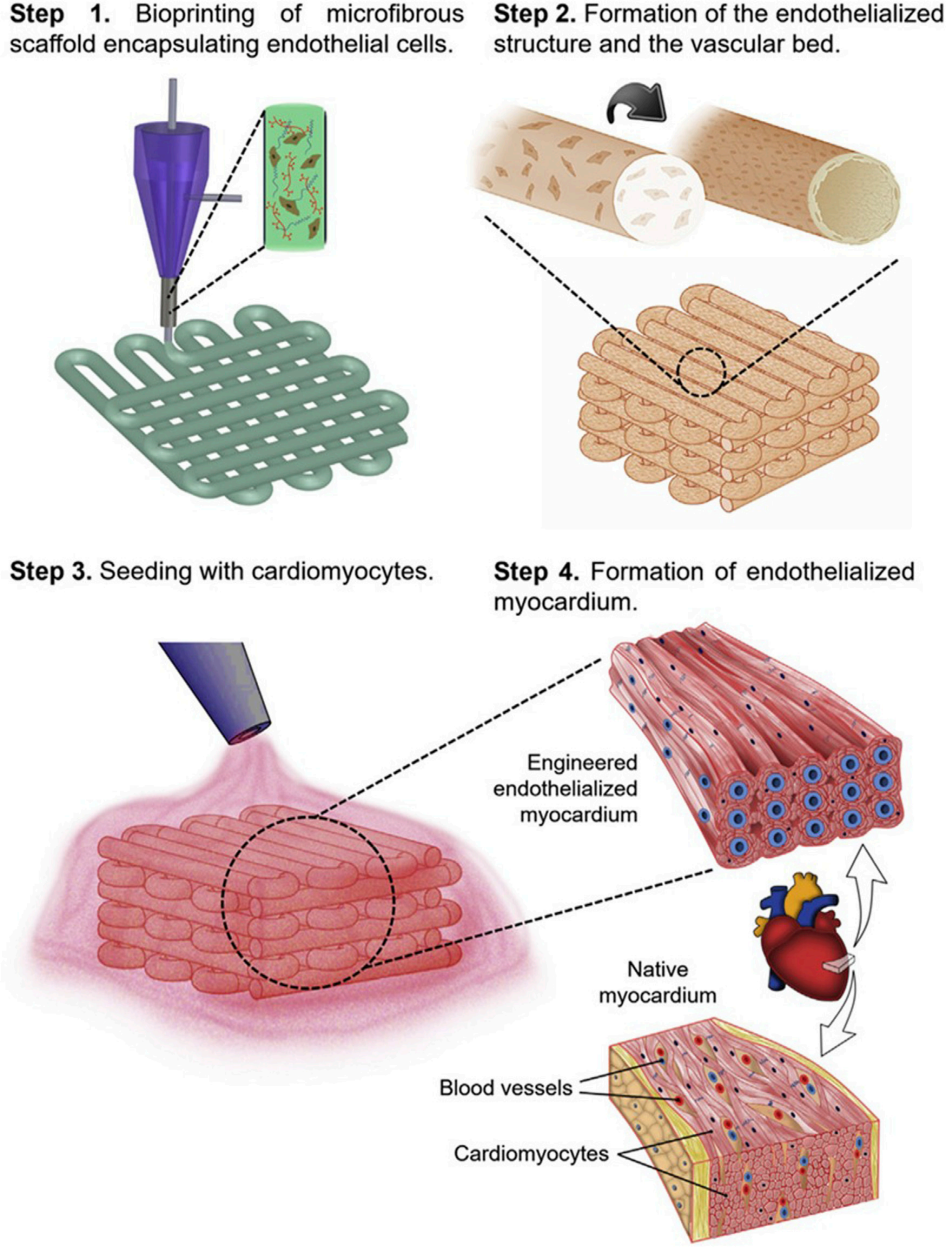
Schematics outlining the application of 3D bioprinting to fabricate an endothelialized myocardium. Step 1: A composite bioink, based on alginate and GelMA and containing endothelial cells, is used to bioprint a microfibrous scaffold. Step 2: Endothelial cells migrate towards the peripheries of the microfibers and create a layer of confluent endothelium. Step 3: The interstitial spaces of the endothelialized scaffold are seeded with cardiomyocytes. Step 4: An engineered endothelialized myocardium, with native tissue-like structure, is obtained. Reprinted from Zhang et al., 2016. Reproduced with permission from Elsevier.

For example, Zhang et al. developed a novel hybrid strategy based on 3D bioprinting, for engineering a heart-on-a-chip (Figure 11) (Zhang et al., 2016). In this study, endothelial cells were bioprinted alongside a bioink composed of a blend of alginate and GelMA, using a combination of extrusion and photocuring techniques. Once the endothelial cells formed a complete endothelial layer, cardiomyocytes were seeded onto the scaffold, leading to the formation of an aligned myocardial tissue capable of spontaneous and synchronized contractions. This resulting organoid was subsequently integrated into a microfluidic perfusion bioreactor, creating an endothelialized-heart-on-a-chip device. This innovative platform was employed to assess the cardiovascular toxicity of doxorubicin, a commonly used anti-cancer drug, and demonstrated dose-dependent effects on both cell types. As a progressive step towards the development of *in vitro* human tissue models, rat cardiomyocytes were substituted with human-induced pluripotent stem cell-derived cardiomyocytes, yielding comparable outcomes. Overall, this study underscores the potential of combining bioprinting, microfluidics, and stem cells as a transformative technology for creating advanced human tissue models of healthy and diseased myocardium, with potential applications in personalized drug screening.

In another paper, Anil Kumar et al. fabricated a cardiac cell-laden construct by 3D bioprinting, using as bioink a visible-light cross-linkable mixture of gelatin and fibrin (Anil Kumar et al., 2019). In this study, the constructs were loaded with either hiPSC-CMs or a combination of human CMs cell lines and adult human cardiac fibroblasts. Notably, the co-culture of CMs and cardiac fibroblasts, which are two of the most critical cell types for studying cellular physiology, was explored for the first time in this research, according to the authors. The printed cells exhibited outstanding viability, proliferation, and heterocellular coupling, which is of utmost importance for maintaining the normal physiology of the cardiac wall. Consequently, these constructs can serve as a highly authentic model for investigating cardiac diseases and conducting drug screening studies.

## 7 Conclusion and future perspectives

In recent decades, CTE has emerged as a promising strategy for treating MI. Given the complex nature of cardiac tissue, the development of biomimetic scaffolds that mimic the composition, microstructure, and multifunctional properties of the native cardiac ECM has been proposed as a promising approach to create engineered tissue that closely resembles the native myocardium in terms of both structure and function. However, despite significant progress, replicating the full complexity of the native myocardial microenvironment remains a continuing challenge in the field of CTE.

This article reviews the most recent advancements in the development of biomimetic strategies for cardiac regeneration, through the combination of natural-origin materials with cutting-edge 3D printing and bioprinting scaffold fabrication techniques and functionalization strategies. As outlined in the previous sections, results reported in the literature in the last 10 years, pointed out that 3D printed natural-polymer based scaffolds can replicate the composition and the architecture of native cardiac ECM more accurately, with respect to traditional scaffold fabrication techniques.

However, this is still not totally satisfactory, as currently available 3D printed cardiac patches are mainly intended to replace the myocardium, but are not able to replicate the complex three-layer structure (comprising the endocardium on the inside, the myocardium in the middle and the epicardium on the outside) of the native heart muscle. This is a limitation, considering that cells of the endocardial and epicardial layers play a critical signaling role in overall cardiac tissue development and organization. Therefore, future generation of 3D printed scaffolds for heart regeneration should recapitulate the three-layer architecture of the heart wall, including also endocardial and epicardial cells, as this could significantly improve the growth, viability and integration with surrounding tissue of the tissue engineered myocardial patch.

Currently available 3D printed cardiac patches are also not totally satisfactory in reproducing the complex biomechanical environment of the cardiac tissue, which include both mechanical anisotropy and auxetic properties. In the native tissue, mechanical anisotropy is given by the preferential orientation of cells and ECM components, which causes different mechanical properties in different directions. Moreover, interstices among cardiac muscle fibers allow volumetric deformation and simultaneous expansion in multiple directions, with a negative Poisson’s ratio, which is indicative of auxetic properties. In particular, while several papers in literature describe the development of cardiac patches with anisotropic mechanical properties, auxetic properties are often overlooked. Therefore, an auxetic design should be considered in developing cardiac patch and 3D printing technique could be useful in producing unique microarchitectures which provide a negative Poisson’s ratio.

The scaffold should also promote tissue growth and organization through the release of bioactive signals, modulate the immune response, guarantee an electromechanical coupling with the surrounding tissue, promote a rapid neovascularization and, ideally, be implantable through a minimally invasive procedure. All of these additional features can be provided to the scaffold through appropriate multi-functionalization strategies. A large number of papers in literature describe promising scaffold functionalization techniques, but most of them have been investigated on traditionally fabricated scaffolds. As described in section 5, only a few papers in literature report the development of functionalized 3D printed natural polymer-based scaffold. In addition, when it is done, only one additional functionality is introduced in the scaffold. According to us, the development of multifunctional 3D printed scaffold should become an important direction for future research.

Bioengineered scaffolds mimicking, in all of their characteristics, the complex natural myocardial tissue, through a synergic combination of different available technologies, would be the key to move CTE from the research arena to routine clinical practice.
